# Supplementary material for: The Plasmodium CSP repeats have elastic properties with a critical role in sporozoite motility
Source: EMBO J. 2025 Sep 22;44(21):6253–72. doi: 10.1038/s44318-025-00551-9 (PMC12583564; doi:10.1038/s44318-025-00551-9)
Supplement: Supplementary file 1 — Appendix [file 44318_2025_551_MOESM1_ESM.pdf]

Appendix for:
"The *Plasmodium* CSP repeats have elastic properties with an important role in sporozoite motility"

Appendix Table S1. Comparison of CSP Repeat Sequences Across *Plasmodium* Species pages 2-3

Appendix Figure S1. Generation and validation of CSP repeat mutants pages 4-5

Appendix Figure S2. Recombinant control (RCon) and WT-GFP sporozoites have similar phenotypes pages 6-7

Appendix Figure S3. CSP Repeat mutants exhibit normal sporozoite development in the mosquito prior to salivary gland invasion. page 8

Appendix Figure S4. TSR exposure in ΔRep2 is not due to enhanced CSP cleavage page 9

Appendix Figure S5. The percentage of CSP repeat mutants that are motile is similar to controls page 10

Appendix Figure S6. Instantaneous speed plots of circular gliding CSP repeat mutants page 11

150 Mammalian Host	Species	CSP Repeat Sequence
Human	***Plasmodium falciparum***	ADG NPDP NANP NVDP NANP NVDP NANP NVDP NANP NANP NANP NANP NANP NANP NANP NANP NANP NANP NANP NANP NANP NANP NANP NANP NANP NANP NVDP NANP NANP NANP NANP NANP NANP NANP NANP NANP NANP NANP NANP NANP NANP NANP NANP NANP NANP
	Plasmodium vivax	GDRA DGQPA GDRA DGQPA GDRA DGQPA GDRA DGQPA GDRA AGQPA GDRA DGQPA GDRA DGQPA GDRA DGQPA GDRA DGQPA GDRA AGQPA GDRA AGQPA GDRA DGQPA GDRA AGQPA GDRA DGQPA GDRA AGQPA GDRA DGQPA GDRA AGQPA GDRA AGQPA GDRA AGQAA GDRA AGQAA GGNAG GQGQ
	Plasmodium malariae	PGDD DGAG NDAG NDAG NAAG NAAG NAAG NAAG NDAG NAAG NAAG NAAG NAAG NAAG NAAG NAAG NAAG NAAG NAAG NAAG NAAG NAAG NAAG NAAG NAAA GNAA GNAA GNAA GAAG NAAG NAAG NAAG NAAG NAAG NAAG NAAG NAAG NAAG NAAG NAAG NAAG NAAG NAAG
	Plasmodium ovale	DRENDP PAPVP QGDP PAPVP QGDP PAAVP QGDP PAPVP QGDP PAPQG DPPA PVPQG DPPA PVPQG DPPA PVPQG NPPA PVPQG DPPA PQGDP PAPV PQGDP PAPA PQGDP PAPA PQGDG KPPA PAPQG DGNQ PAPG
Rodent	***Plasmodium berghei***	PPPP NPNDPPPP NPNDPPPP NPNDPPPP NPNDPPPP NANDPPPP NANDPAPP NANDPAPP NANDPAPP NANDPAPP NANDPPPP NPNDPAPP NANDPPPP NPNDPAPP QGNNN PQPQ PRPQ PQPQ PQPQ PQPQ PQPQ PRPQ PQPQ P
	Plasmodium yoelii	VVADENVDQ GPGAPQ E PPQQ PPQQ PPQQ PPQQ PPQQ PPQQ PPQQ P
	Plasmodium chabaudi	GGQGGQ GVQGDQ GGQGVQ GDQGGQ GVQGDQ GGQGVQ GDQGGQ GVQGDQ GGQGVQ GDQGGQ GVQGDQ GGQGVQ GDQGGQ GVQGDQ GGQGGQ GGQGAQ GAQGGQ GGQGVQ GDQGGQ GGQGGQ GGQGGQ GGQGGQ GDQGAQ GADGNR
Non-Human Primate	***Plasmodium knowlsei***	NE GQPQAQ GDGANA GQPQAQ GDGANA GQPQAQ GDGANA GQPQAQ GDGANA GQPQAQ GDGANA GQPQAQ GDGANA GQPQAQ GDGANA GQPQAQ GDGANA GQPQAQ GDGANA GQPQAQ GDGANA GQPQAQ GDRANA GQPQAQ GDGANV
	Plasmodium cynomolgi	VENAD GNAG GNAG GNAG GNAG GNAG GNAD GNAG GNAG GNAG GNAG GNAG GNAD GNAG GNAG GNAG GNAD GNAG GNAG GNAG GNAD GNAG GNAG GNAG GNAG GNAD GNAG GNAG GNAD GNAG GNAG GNAG GNAD GNAG GNAG GNAG GNAG GNAG GNAD GNAG GNAG GNAG GNAG GNAG GNAG GNAG GNAG GNAG GNAG GNAG GNAG ANAGN

Appendix Table S1. Comparison of CSP Repeat Sequences Across *Plasmodium* Species. Within each sequence the individual repeat blocks are separated for illustrative purposes. We define the repeat region as beginning just after the highly conserved Region I: In most cases there is an initial 3 to 9 amino acid non-repetitive sequence as shown. Sequences are from the May 2024 release of PlasmoDB (https://plasmodb.org/plasmo/app). Gene IDs used for these sequences are: PfNF54_030009700, PVX_119355, PmUG01_08051600, POWCR01_000063800, PBANKA_0403200, PY17X_0405400, PCHAS_0404100, PKNH_0838500, PcyM_0839700.

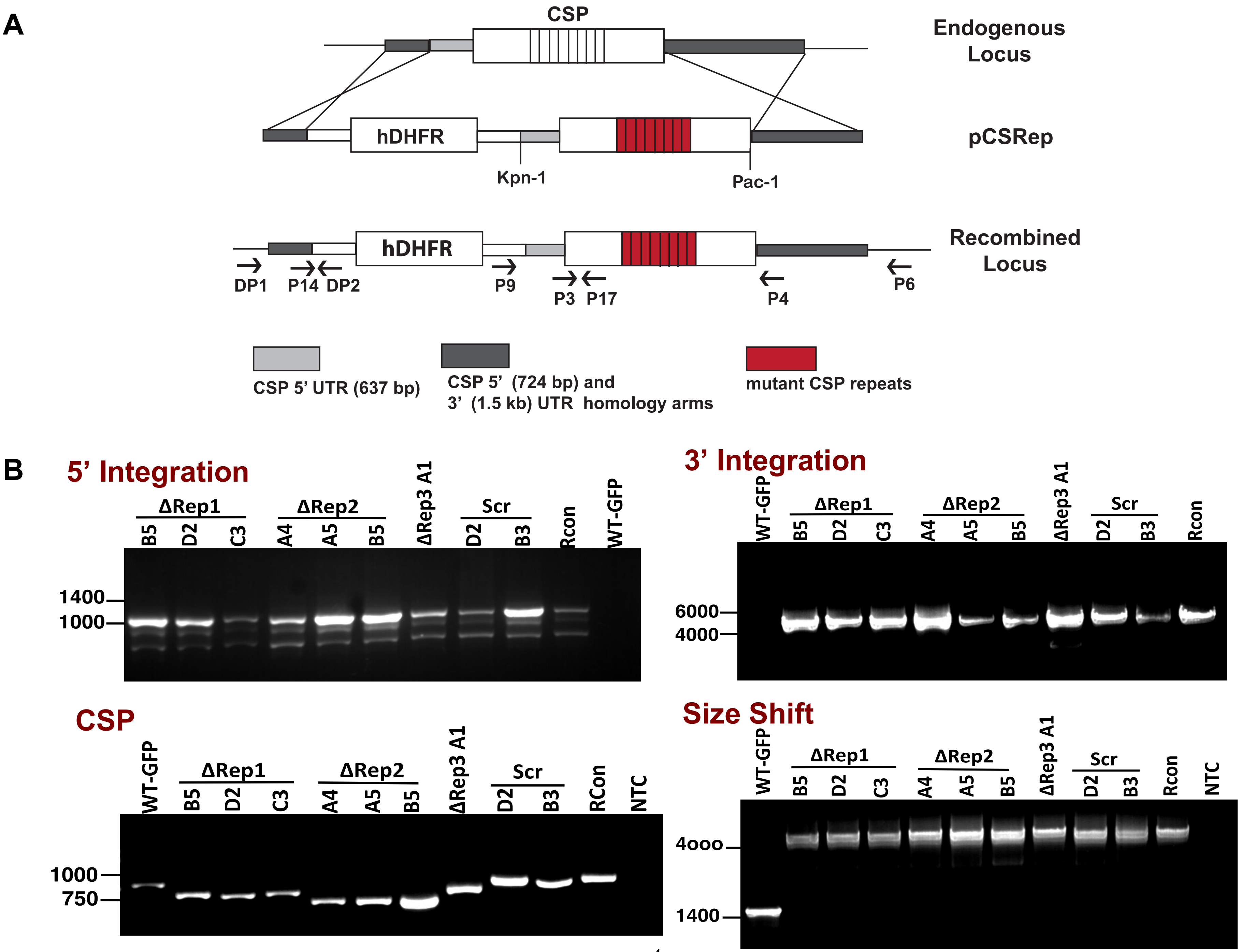
A
CSP
Endogenous Locus
hDHFR
pCSRep
Kpn-1
Pac-1
hDHFR
Recombined Locus
DP1
P14 DP2
P9
P3 P17
P4
P6
CSP 5' UTR (637 bp)
CSP 5' (724 bp) and 3' (1.5 kb) UTR homology arms
mutant CSP repeats
B
5' Integration
ΔRep1
B5 D2 C3
ΔRep2
A4 A5 B5
ΔRep3 A1
Scr
D2 B3
Rcon
WT-GFP
1400
1000
3' Integration
WT-GFP
ΔRep1
B5 D2 C3
ΔRep2
A4 A5 B5
ΔRep3 A1
Scr
D2 B3
Rcon
6000
4000
CSP
WT-GFP
ΔRep1
B5 D2 C3
ΔRep2
A4 A5 B5
ΔRep3 A1
Scr
D2 B3
RCon
NTC
1000
750
Size Shift
WT-GFP
ΔRep1
B5 D2 C3
ΔRep2
A4 A5 B5
ΔRep3 A1
Scr
D2 B3
Rcon
NTC
4ooo
1400

Appendix Figure S1. Generation and validation of CSP repeat mutants. (**A**) Transfections were performed to replace the endogenous *csp* locus with a *csp* gene that contained a repeat region with the desired mutations. The indicated fragment from pCSRep was released from the plasmid by restriction digest, with the 5' and 3' UTR homology arms driving homologous recombination to give rise to the recombinant locus shown. The hDHFR selection cassette upstream of *csp* enabled positive selection of recombinant parasites. Binding sites of primers described in the genotyping panels below are also shown. (**B**) Clonal lines were verified using a series of PCR reactions: DP1 and DP2 for 5' integration, P9 and P6 for 3' integration; P3 and P4 for the *csp* gene with size changes due to truncation and P14 and P17 which shows a size shift in recombinant parasites due to the insertion of the hDHFR cassette. NTC, no template control.

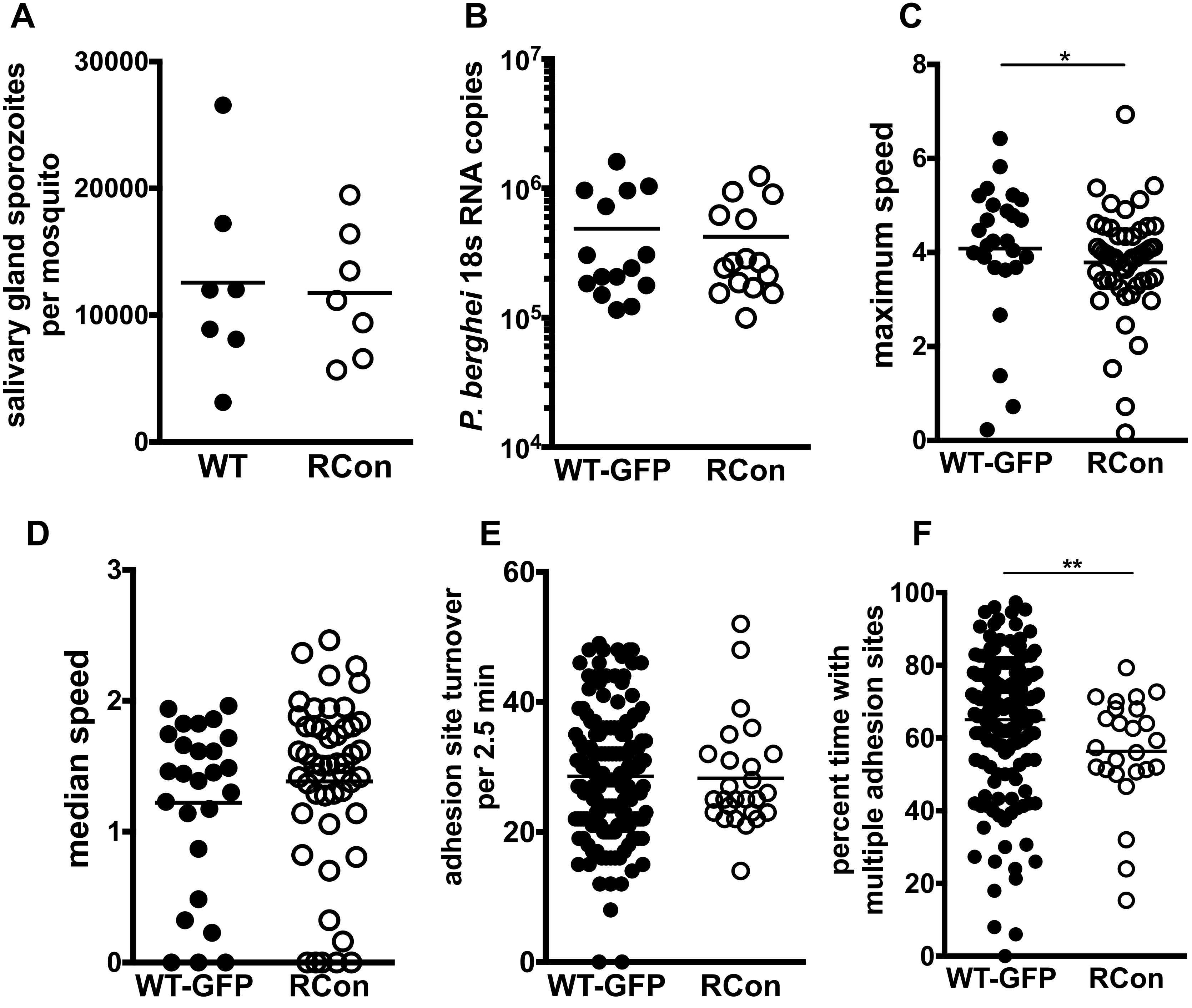
A
salivary gland sporozoites per mosquito
30000
20000
10000
0
WT
RCon
B
P. berghei 18s RNA copies
10^7
10^6
10^5
10^4
WT-GFP
RCon
C
maximum speed
*
8
6
4
2
0
WT-GFP
RCon
D
median speed
3
2
1
0
WT-GFP
RCon
E
adhesion site turnover per 2.5 min
60
40
20
0
WT-GFP
RCon
F
percent time with multiple adhesion sites
**
100
80
60
40
20
0
WT-GFP
RCon

Appendix Figure S2. Recombinant control (RCon) and WT-GFP sporozoites have similar phenotypes. (**A**) Salivary gland sporozoite loads. Salivary glands from 20 infected mosquitoes were pooled, homogenized and the average number of sporozoites per mosquito was determined. Each data point is from an independent mosquito cycle with matched WT-GFP and RCon lines and data are pooled from 7 biological replicates. Paired Wilcoxon t-test at $p<0.05$ showed no statistically significant difference. Welch's t-test $p = 0.8168$. (**B**) Sporozoite liver infection. 5,000 WT-GFP or RCon sporozoites were inoculated IV into C57Bl/6 mice and 40 hours later, parasite liver burden was determined by RT-qPCR using primers specific *P. berghei* 18s RNA. Shown are pooled data from 3 experiments with 5 mice per group in each experiment. (**C&D)** Live gliding assay showing maximum (C) and median (D) sporozoite speeds from 26 (WT-GFP) and 50 (RCon) sporozoites. For (C): Mann-Whitney-Wilcoxon *p=0.047. For (D): Welch's t-test $p = 0.3036$. (**E**&**F**) Adhesion site analysis using RICM. RICM movies of circular gliding WT-GFP and RCon sporozoites were analyzed to quantify the frequency with which adhesion sites move from the sporozoite's anterior to posterior end (E) and the frequency with which gliding sporozoites have multiple adhesion sites (F), Mann-Whitney-Wilcoxon **p = 0.004. All movies included 2.5 minutes of circular gliding for analysis. Data are pooled from at least 3 independent experiments with the following number of sporozoites per line: WT-GFP=175 and RCon = 25.

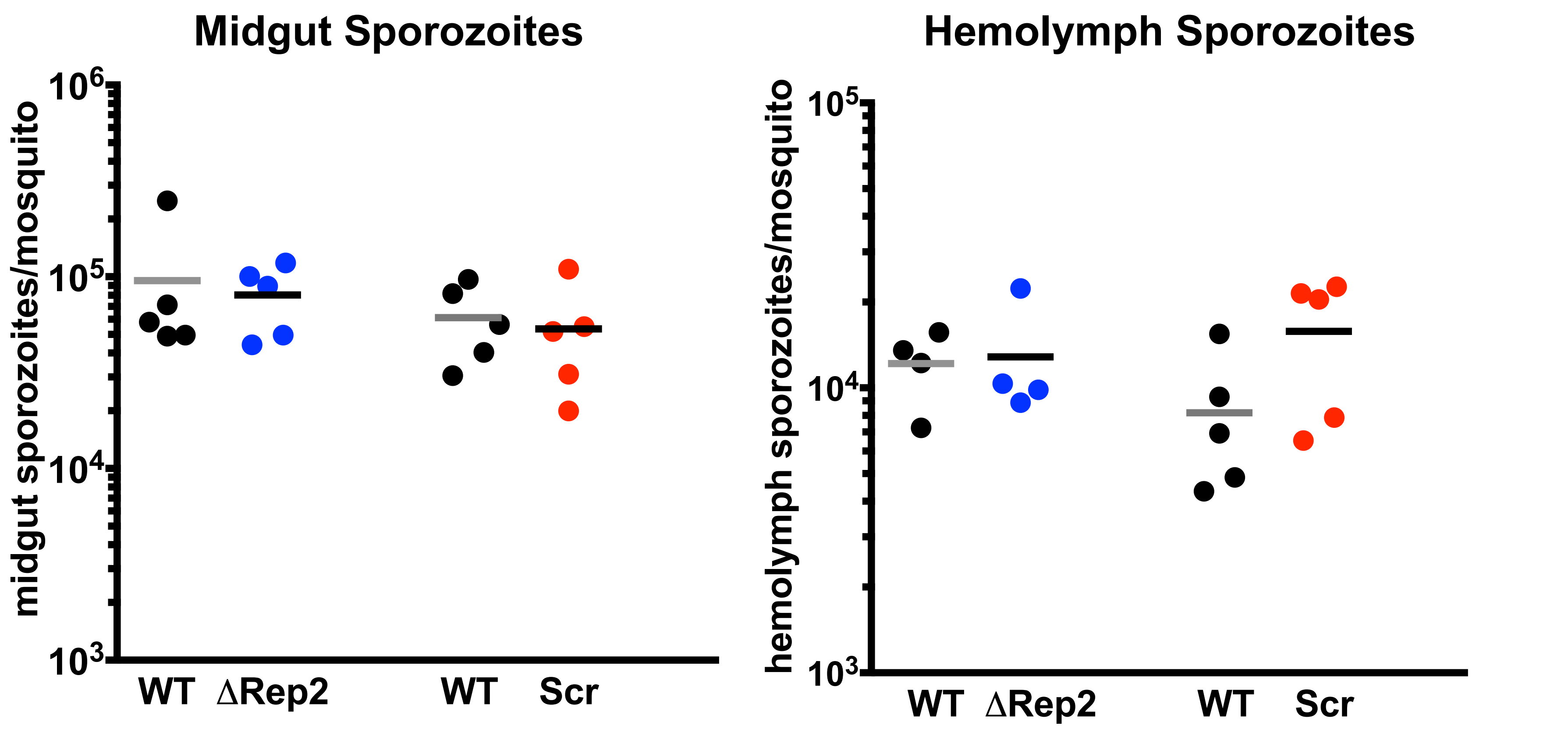

Appendix Figure S3. CSP Repeat mutants exhibit normal sporozoite development in the mosquito prior to salivary gland invasion. Midgut (left panel) and hemolymph (right panel) sporozoites were collected from 20 mosquitoes and counted. Shown is the average number of midgut or hemolymph sporozoites per mosquito. Each data point is from an independent mosquito cycle with matched control lines and shown are pooled data from 5 to 6 biological replicates. Kruskal-Wallis showed no significant differences between mutant lines and matched controls (midgut sporozoites, p=0.6026; hemolymph sporozoites, p=0.2979).

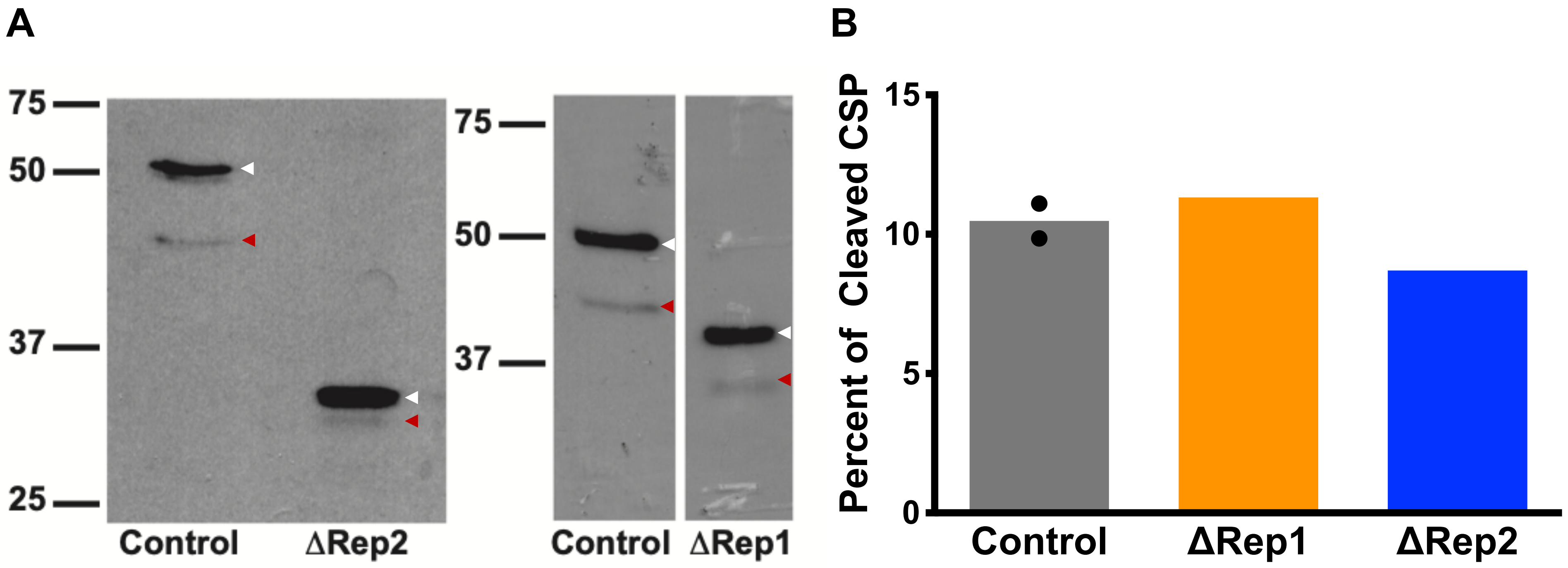

Appendix Figure S4. TSR exposure in ΔRep2 is not due to enhanced CSP cleavage. (A) Control, ΔRep2, and ΔRep1 sporozoites were metabolically labeled with [^{35}S]-Cys/Met and CSP was immunoprecipitated and run on an SDS-PAGE gel followed by autoradiography. Metabolic label is initially incorporated into full-length CSP (top band, white arrowhead) and over time a small amount of cleaved CSP can be observed (faint bottom band, red arrowhead). (**B**) Band intensities were measured by densitometry and percent of total CSP detected in the bottom band (cleaved) is expressed in the graph in panel B. Densitometry measures of control parasites are combined from both blots in panel A (n=2, with the mean shown by the bar), and for ΔRep1 and ΔRep2 are from the one sample shown in panel A (n=1)

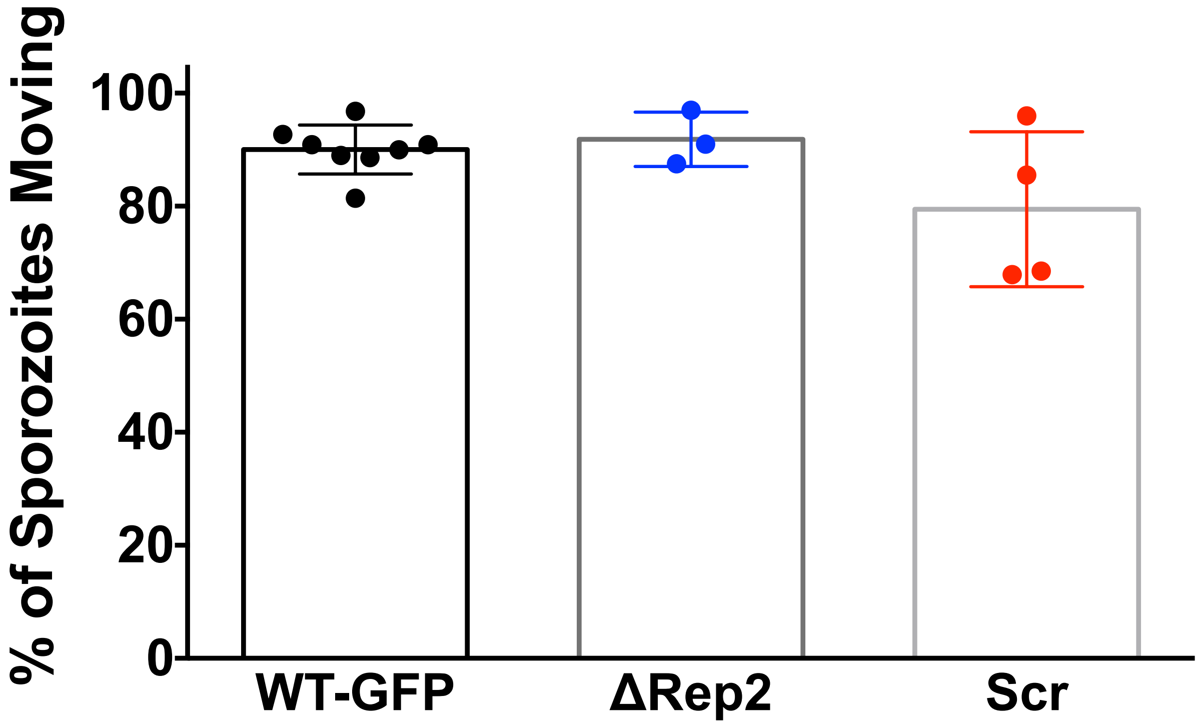

Appendix Figure S5. The percentage of CSP repeat mutants that are motile is similar to controls. Sporozoites were prepared for live gliding experiments, added to coverslips and at least 50 sporozoites per coverslip were counted. Each sporozoite was recorded as moving (circular gliding, patch gliding or attached waving) or not moving (drifting or attached with no movement). Any attached sporozoite that was not moving was observed for at least 30 seconds prior to being recorded as non-motile. Data are pooled from 3 to 4 biological replicates with each dot representing the percentage of sporozoites moving from one experiment with pooled controls that were performed alongside mutants. Shown are the mean +/- standard deviation. Comparison of percent motile between the control and ΔRep2 or Scr line was not significant; Kruskal-Wallis test p=0.2649.

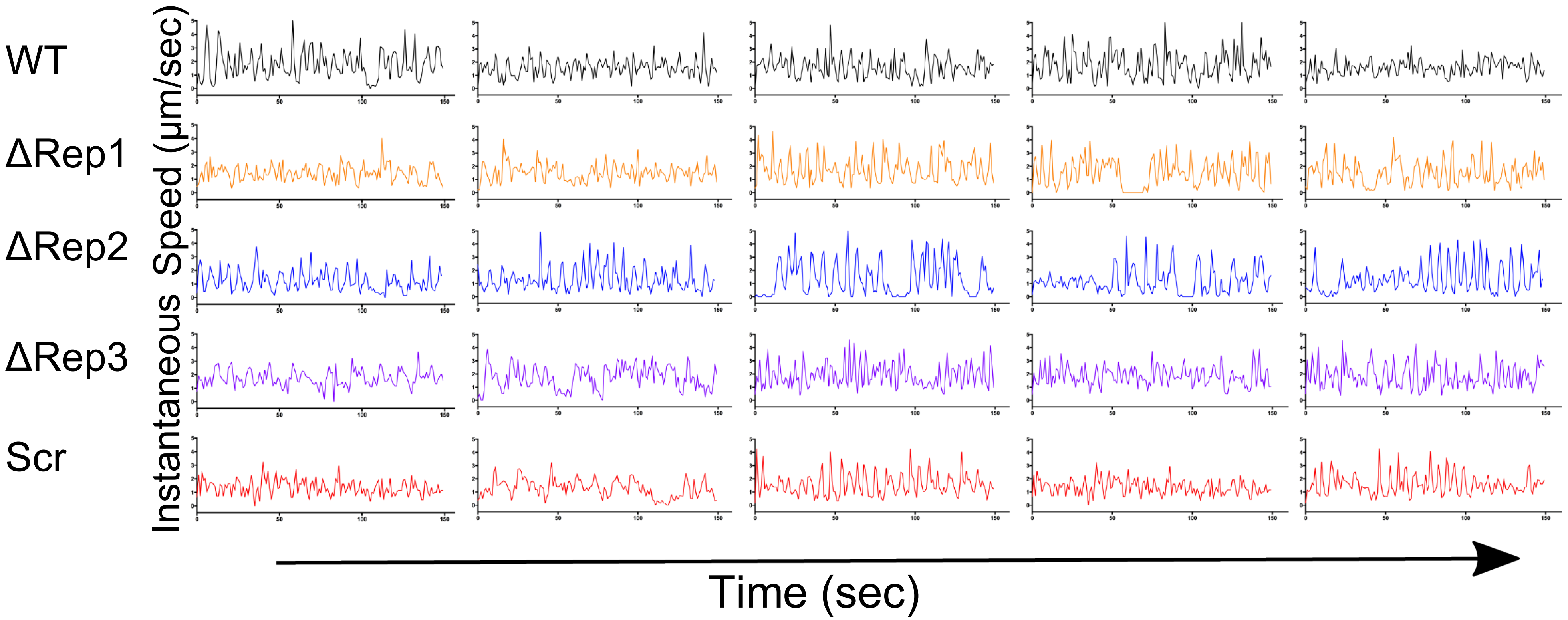

Appendix Figure S6. Instantaneous speed plots of circular gliding CSP repeat mutants. Five representative instantaneous speed plots are shown. Plots were chosen such that each had a median speed close to the mutant's overall median speed (shown in Figure 5B). Both x- and y-axes are identical for all plots.
