# Supplementary material for: The Plasmodium CSP repeats have elastic properties with a critical role in sporozoite motility
Source: EMBO J. 2025 Sep 22;44(21):6253–72. doi: 10.1038/s44318-025-00551-9 (PMC12583564; doi:10.1038/s44318-025-00551-9)
Supplement: Supplementary file 5 — Movie EV3 [file 44318_2025_551_MOESM5_ESM.zip › Movie EV3.docx]

Movie EV3_Rep2_Waving
