# Supplementary material for: The Plasmodium CSP repeats have elastic properties with a critical role in sporozoite motility
Source: EMBO J. 2025 Sep 22;44(21):6253–72. doi: 10.1038/s44318-025-00551-9 (PMC12583564; doi:10.1038/s44318-025-00551-9)
Supplement: Supplementary file 7 — Movie EV5 [file 44318_2025_551_MOESM7_ESM.zip › Movie EV5.docx]

Movie EV5_RICM Rep2 Patch Gliding
