# Supplementary figures and images for: The Plasmodium CSP repeats have elastic properties with a critical role in sporozoite motility
Source: EMBO J. 2025 Sep 22;44(21):6253–72. doi: 10.1038/s44318-025-00551-9 (PMC12583564; doi:10.1038/s44318-025-00551-9)

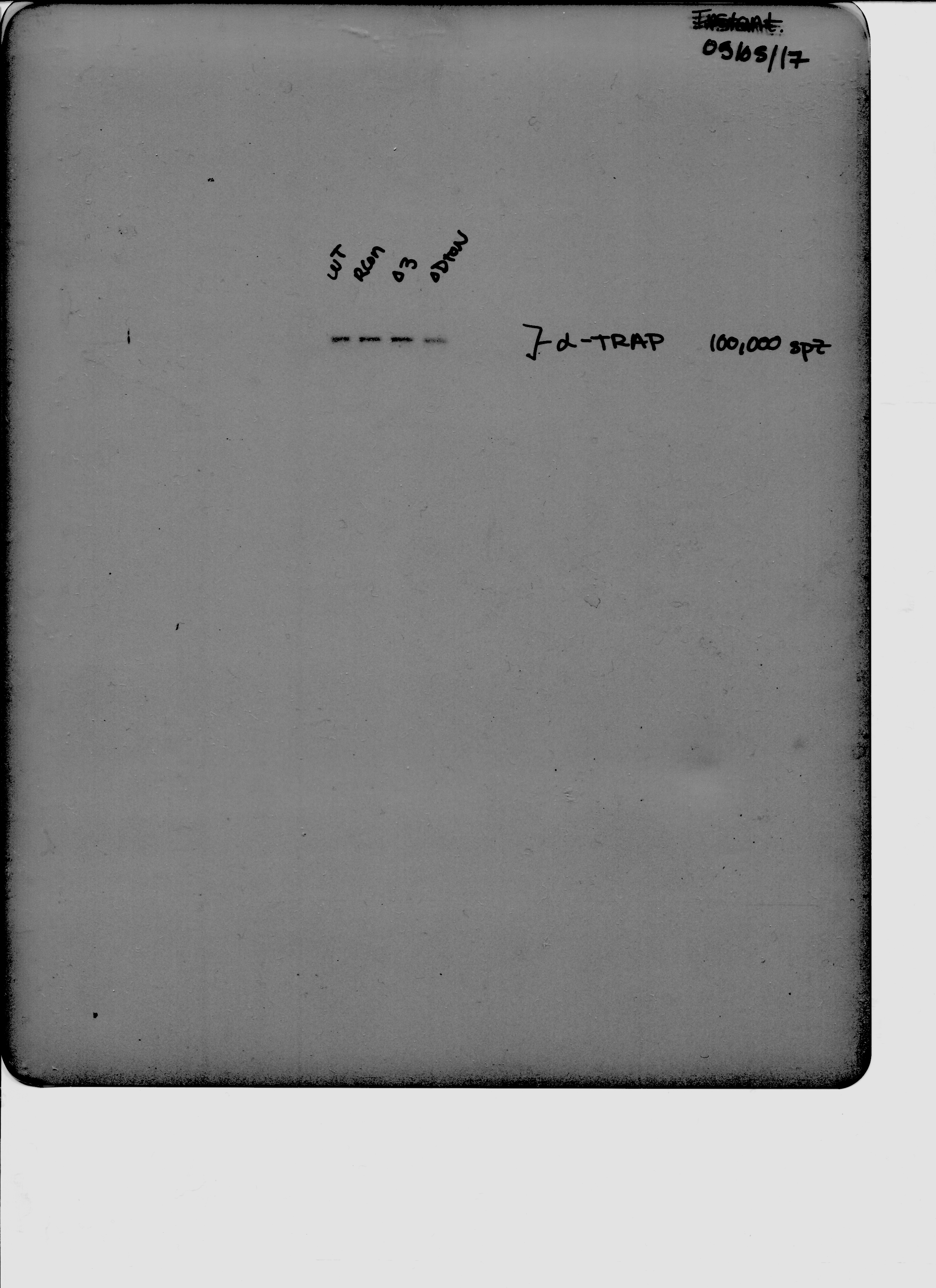

Supplement: Supplementary file 8 — Source data Fig. 1 [file 44318_2025_551_MOESM8_ESM.zip › SD_Figure1_Rev2/Figure1B_Westerns/Fig1B_Rep3_SG_MediumExposureForTRAP.tif]

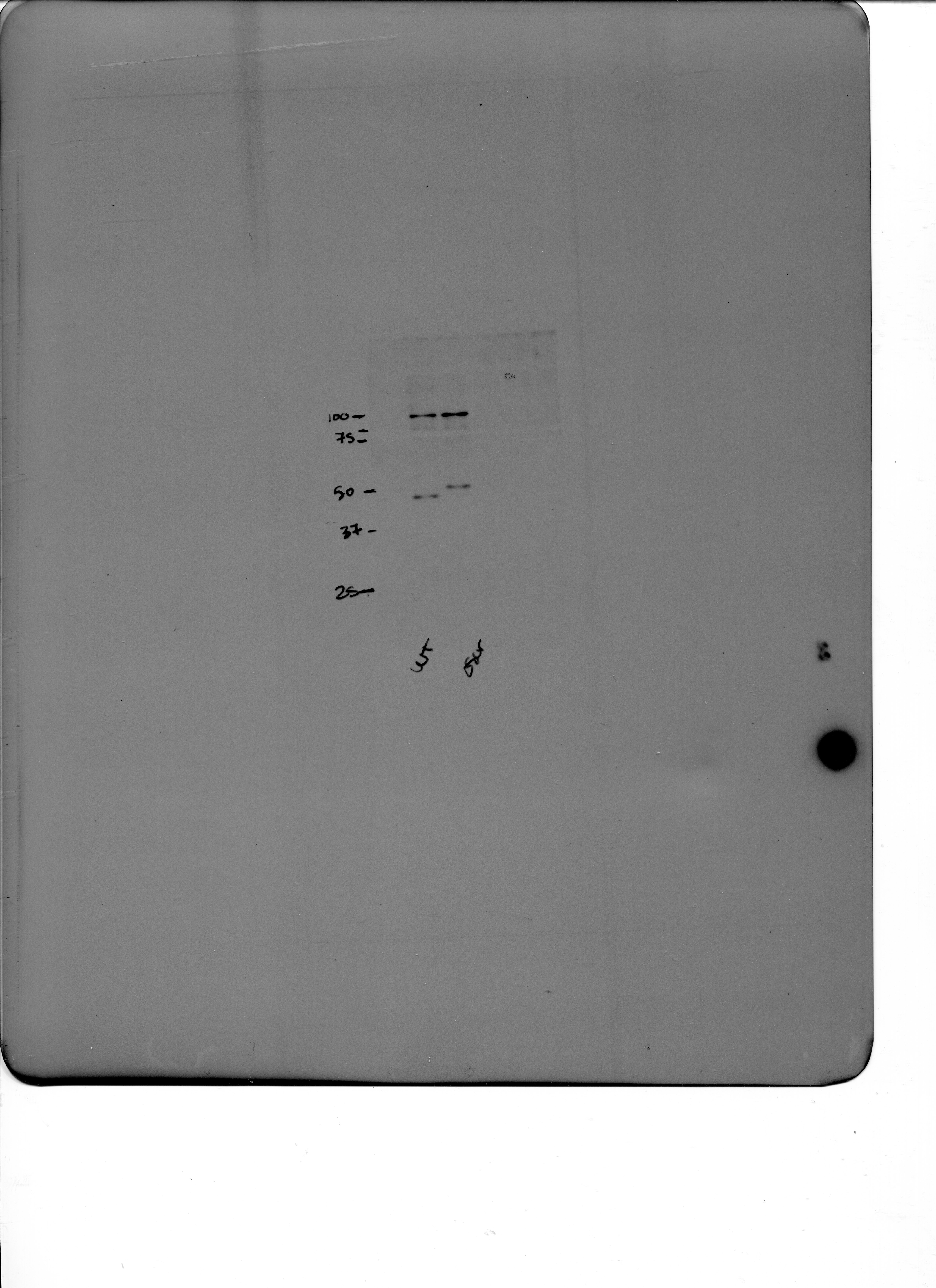

Supplement: Supplementary file 8 — Source data Fig. 1 [file 44318_2025_551_MOESM8_ESM.zip › SD_Figure1_Rev2/Figure1B_Westerns/Fig1B_SCR_HemolymphSpz.jpg]

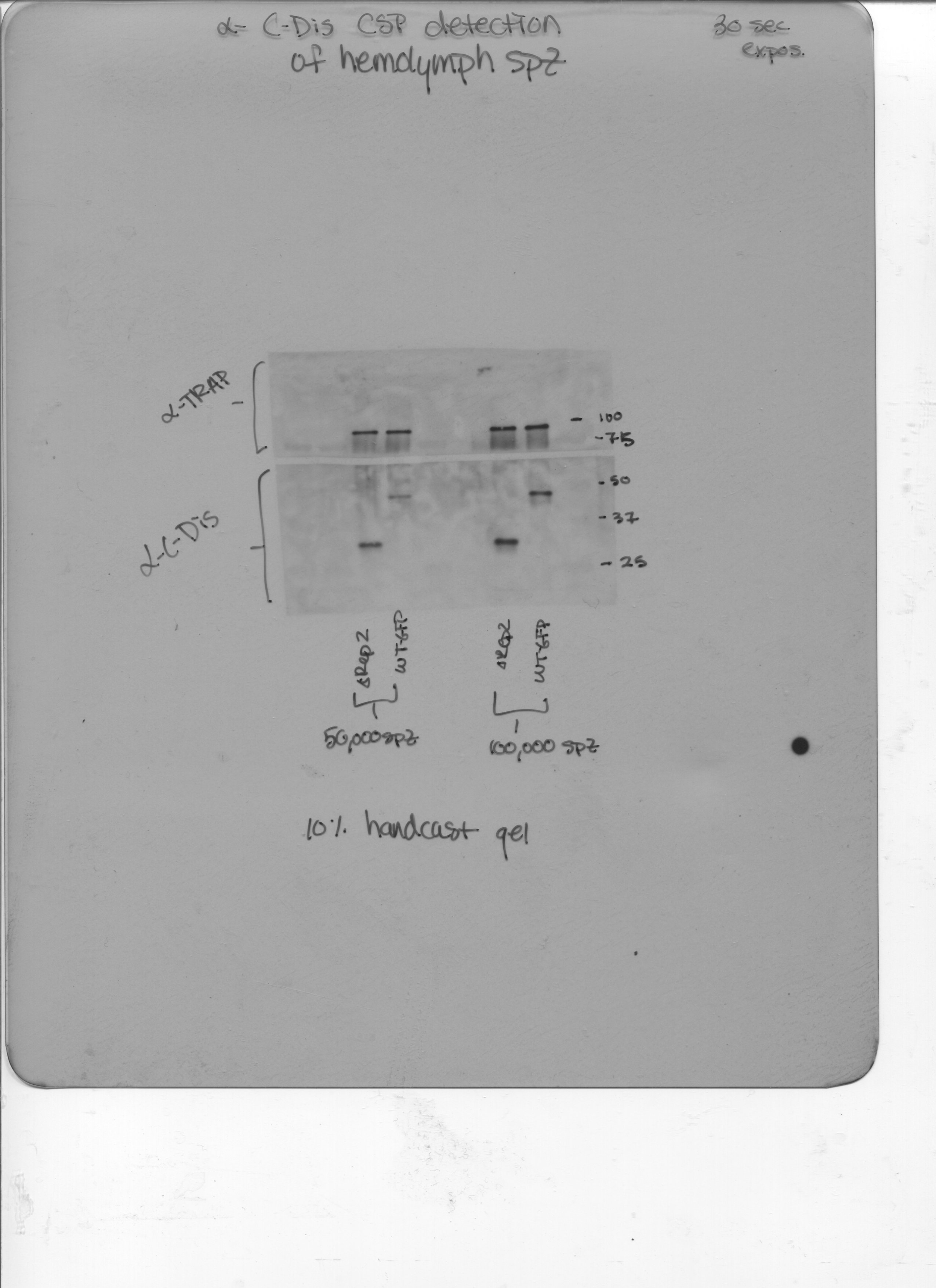

Supplement: Supplementary file 8 — Source data Fig. 1 [file 44318_2025_551_MOESM8_ESM.zip › SD_Figure1_Rev2/Figure1B_Westerns/Fig1B_Rep2 HemolymphSpz.jpg]

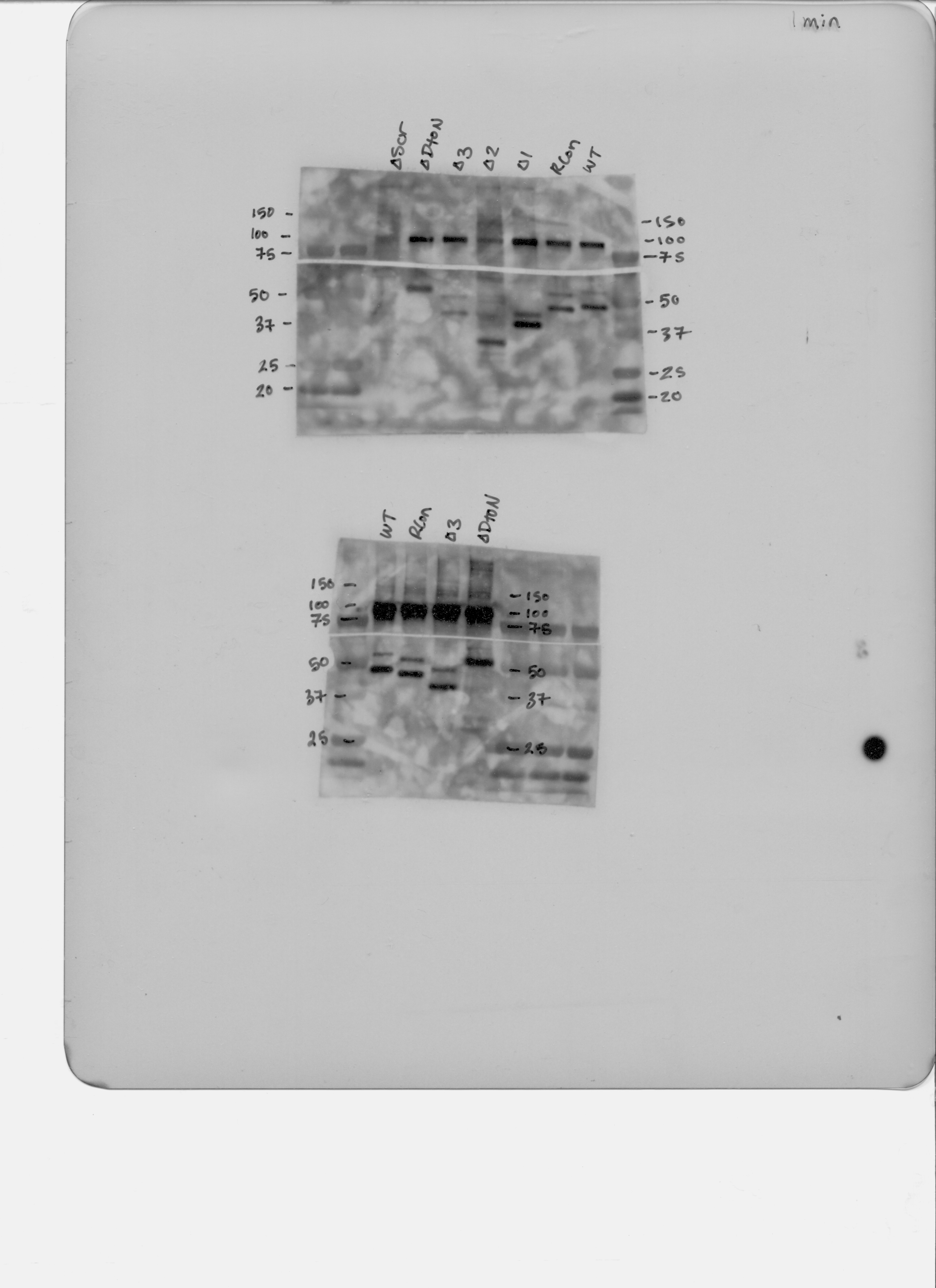

Supplement: Supplementary file 8 — Source data Fig. 1 [file 44318_2025_551_MOESM8_ESM.zip › SD_Figure1_Rev2/Figure1B_Westerns/Fig1B_Rep1&Rep3_SalivaryGlandSpzMediumExposure.tif]

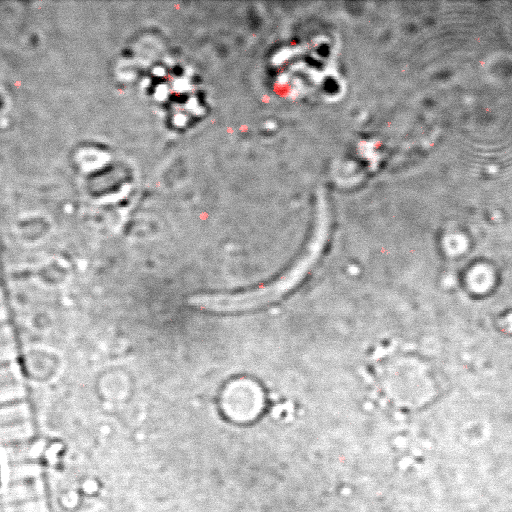

Supplement: Supplementary file 9 — Source data Fig. 2 [file 44318_2025_551_MOESM9_ESM.zip › SD_Figure2_Rev2/Figure2A&B_ImageData/Figure2A_Control_Merge.tif]

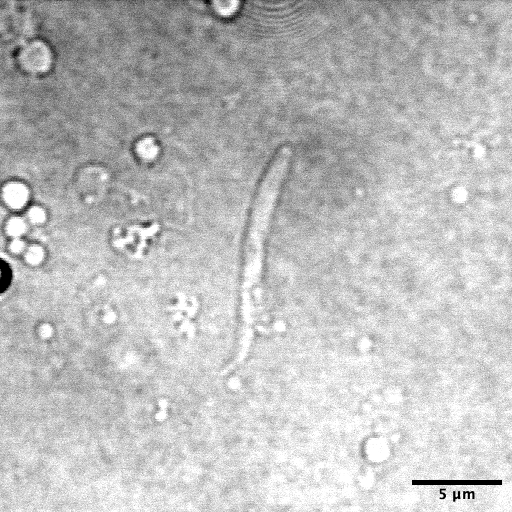

Supplement: Supplementary file 9 — Source data Fig. 2 [file 44318_2025_551_MOESM9_ESM.zip › SD_Figure2_Rev2/Figure2A&B_ImageData/Figure2B_Rep2_DIC.tif]

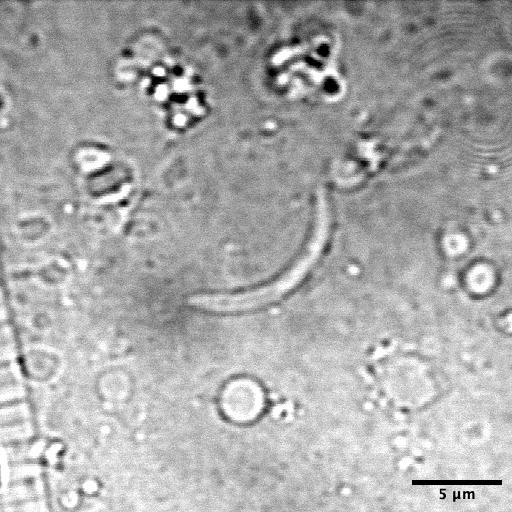

Supplement: Supplementary file 9 — Source data Fig. 2 [file 44318_2025_551_MOESM9_ESM.zip › SD_Figure2_Rev2/Figure2A&B_ImageData/Figure2A_Control_DIC.tif]

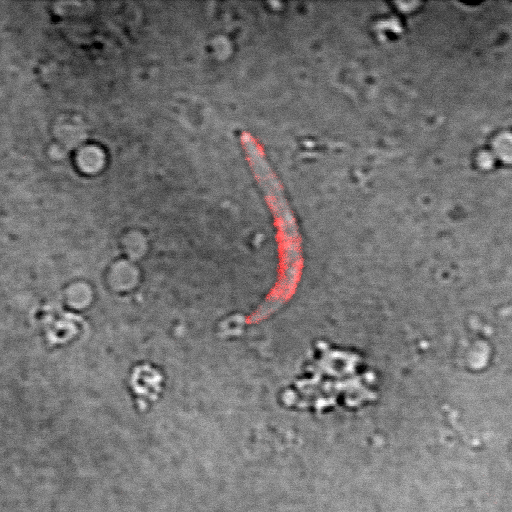

Supplement: Supplementary file 9 — Source data Fig. 2 [file 44318_2025_551_MOESM9_ESM.zip › SD_Figure2_Rev2/Figure2A&B_ImageData/Figure2B_Control_Merge.tif]

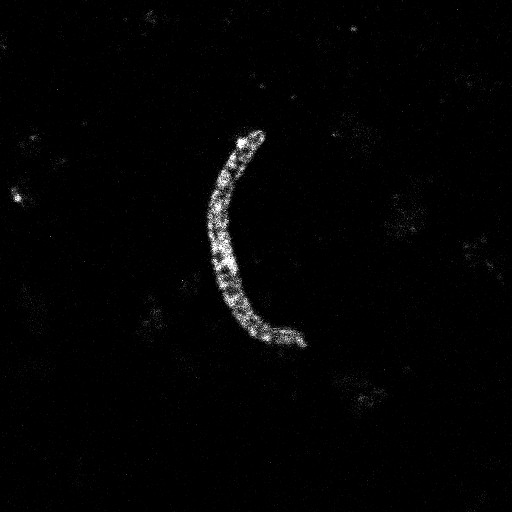

Supplement: Supplementary file 9 — Source data Fig. 2 [file 44318_2025_551_MOESM9_ESM.zip › SD_Figure2_Rev2/Figure2A&B_ImageData/Figure2A_Rep2_Anti-C.tif]

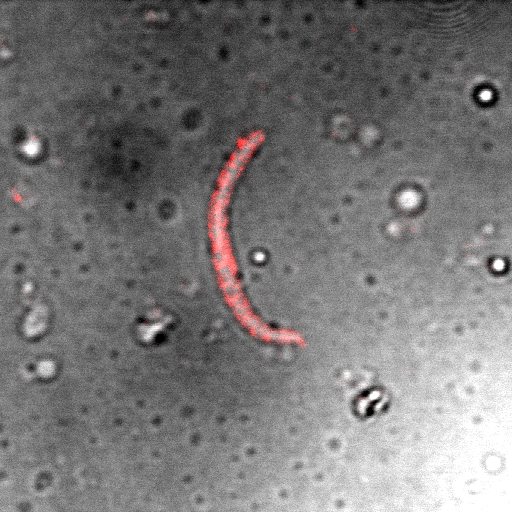

Supplement: Supplementary file 9 — Source data Fig. 2 [file 44318_2025_551_MOESM9_ESM.zip › SD_Figure2_Rev2/Figure2A&B_ImageData/Figure2A_Rep2_Merge.tif]

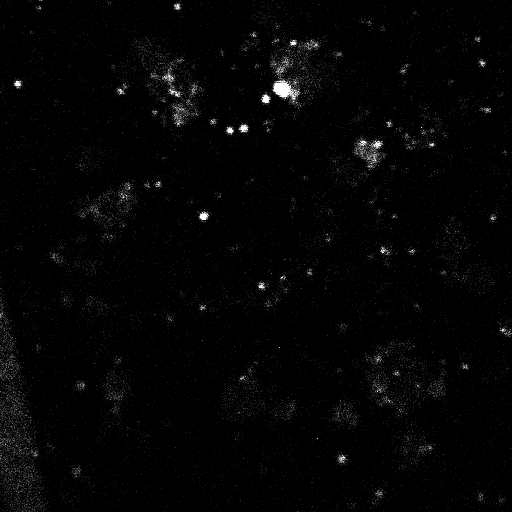

Supplement: Supplementary file 9 — Source data Fig. 2 [file 44318_2025_551_MOESM9_ESM.zip › SD_Figure2_Rev2/Figure2A&B_ImageData/Figure2A_Control_anti-C.tif]

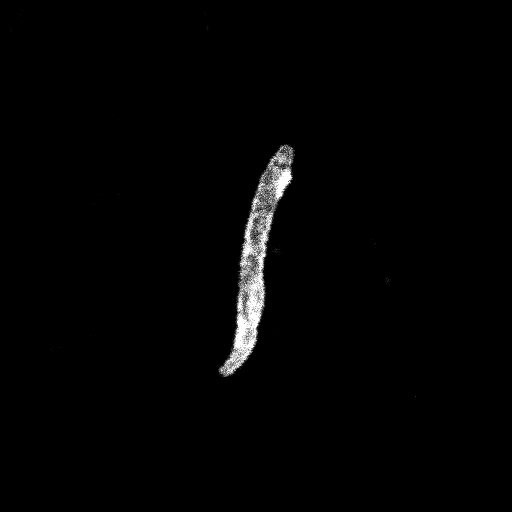

Supplement: Supplementary file 9 — Source data Fig. 2 [file 44318_2025_551_MOESM9_ESM.zip › SD_Figure2_Rev2/Figure2A&B_ImageData/Figure2B_Rep2_Anti-C.tif]

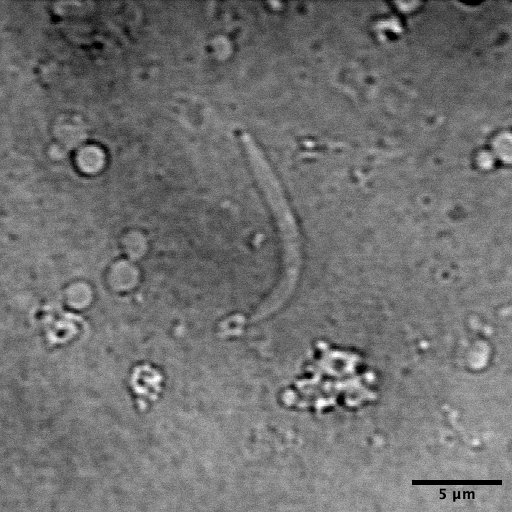

Supplement: Supplementary file 9 — Source data Fig. 2 [file 44318_2025_551_MOESM9_ESM.zip › SD_Figure2_Rev2/Figure2A&B_ImageData/Figure2B_Control_DIC.tif]

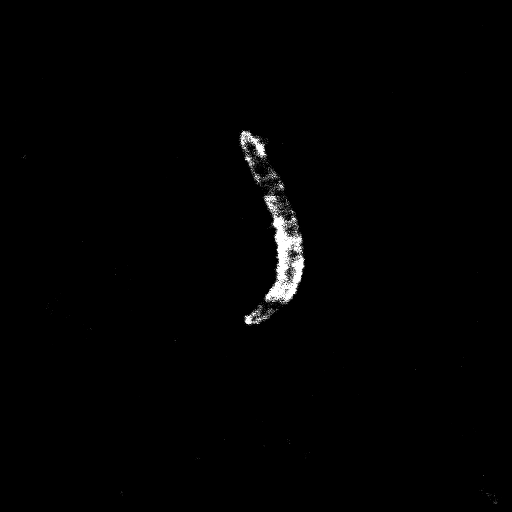

Supplement: Supplementary file 9 — Source data Fig. 2 [file 44318_2025_551_MOESM9_ESM.zip › SD_Figure2_Rev2/Figure2A&B_ImageData/Figure2B_Control_Anti-C.tif]

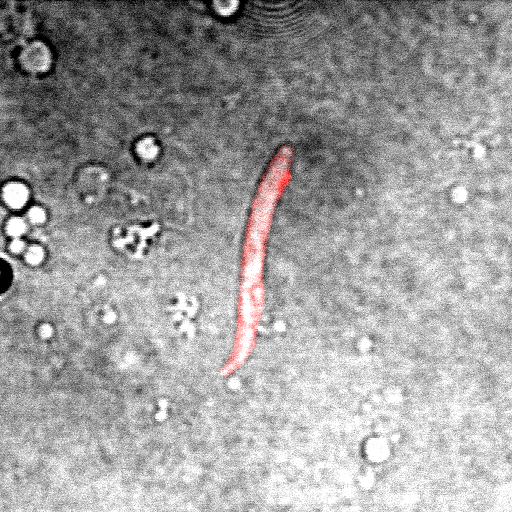

Supplement: Supplementary file 9 — Source data Fig. 2 [file 44318_2025_551_MOESM9_ESM.zip › SD_Figure2_Rev2/Figure2A&B_ImageData/Figure2B_Rep2_Merge.tif]

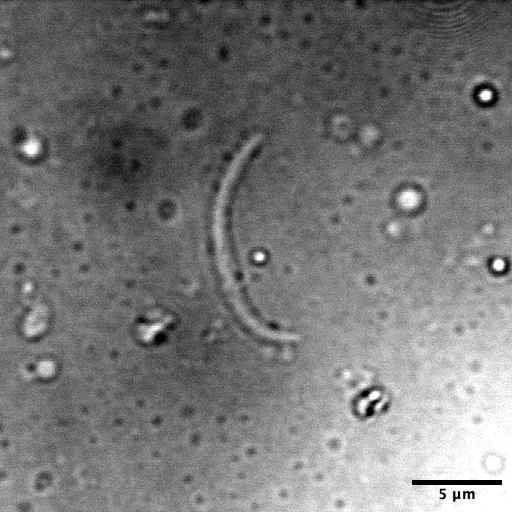

Supplement: Supplementary file 9 — Source data Fig. 2 [file 44318_2025_551_MOESM9_ESM.zip › SD_Figure2_Rev2/Figure2A&B_ImageData/Figure2A_Rep2_DIC.tif]

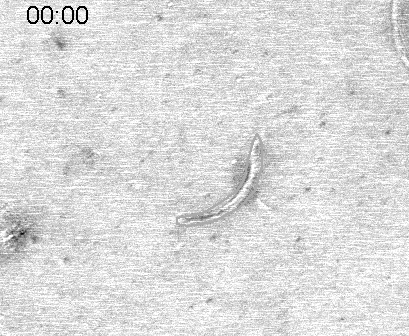

Supplement: Supplementary file 13 — Source data Fig. 6 [file 44318_2025_551_MOESM13_ESM.zip › SD_Figure6_Rev2/Figure6A_ImageData/Figure6A_CircularGliding_RICM_Movie.tif]

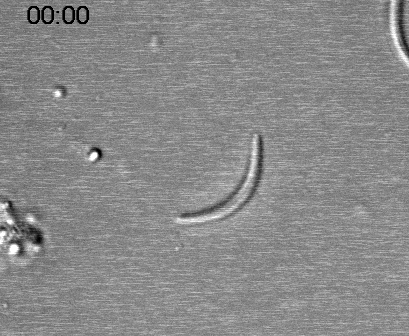

Supplement: Supplementary file 13 — Source data Fig. 6 [file 44318_2025_551_MOESM13_ESM.zip › SD_Figure6_Rev2/Figure6A_ImageData/Figure6A_CircularGliding_DIC_Movie.tif]

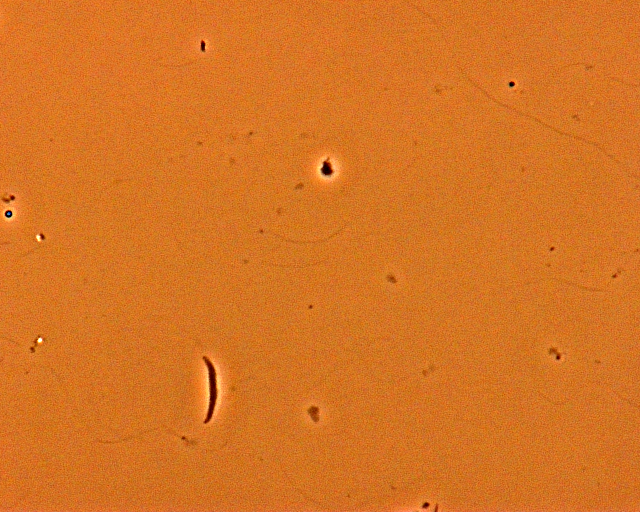

Supplement: Supplementary file 14 — Source data Fig. 7 [file 44318_2025_551_MOESM14_ESM.zip › SD_Figure7_Rev2/Figure7B_ImageData/Figure7B_23-1_Control_Phase.tif]

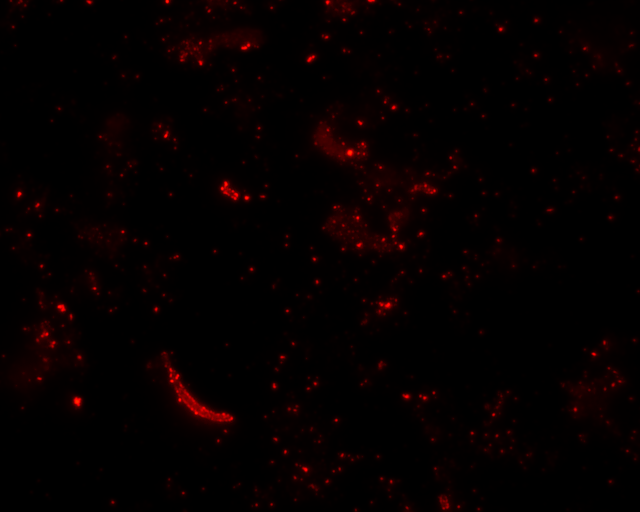

Supplement: Supplementary file 14 — Source data Fig. 7 [file 44318_2025_551_MOESM14_ESM.zip › SD_Figure7_Rev2/Figure7B_ImageData/Figure7B_24-2_Rep2_Fluorescence.tif]

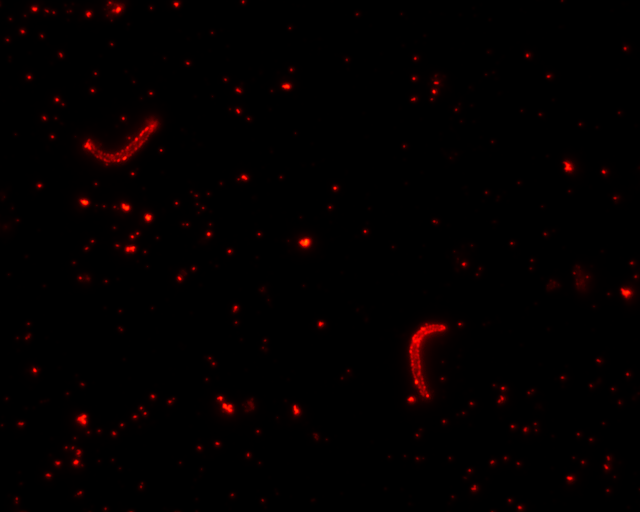

Supplement: Supplementary file 14 — Source data Fig. 7 [file 44318_2025_551_MOESM14_ESM.zip › SD_Figure7_Rev2/Figure7B_ImageData/Figure7B_13-2_Scr_Flourescence.tif]

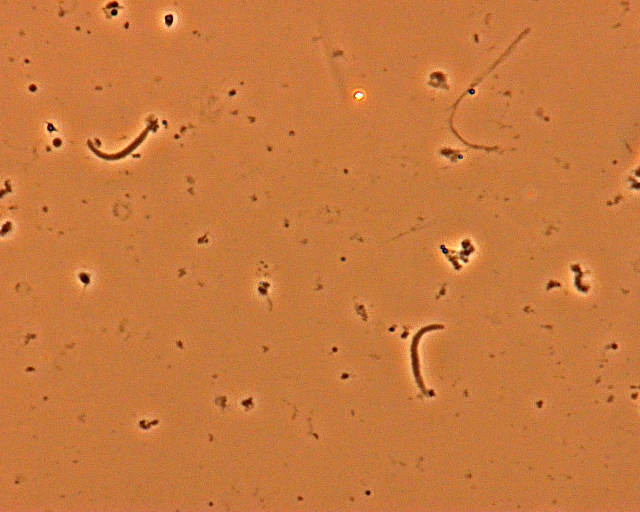

Supplement: Supplementary file 14 — Source data Fig. 7 [file 44318_2025_551_MOESM14_ESM.zip › SD_Figure7_Rev2/Figure7B_ImageData/Figure7B_13-1_Scr_Phase.tif]

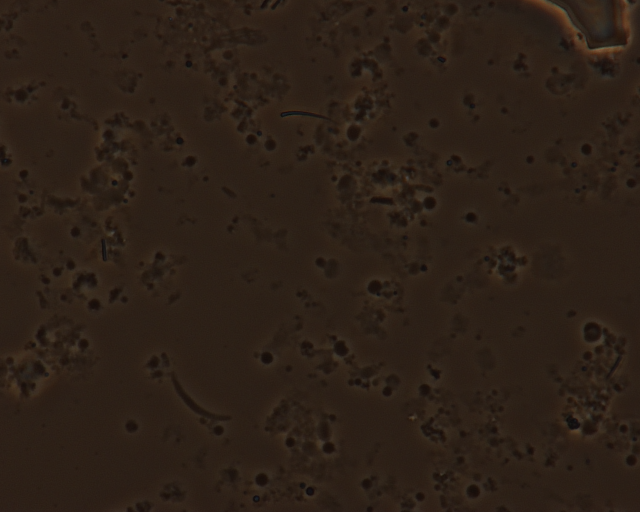

Supplement: Supplementary file 14 — Source data Fig. 7 [file 44318_2025_551_MOESM14_ESM.zip › SD_Figure7_Rev2/Figure7B_ImageData/Figure7B_24-1_Rep2_Phase.tif]

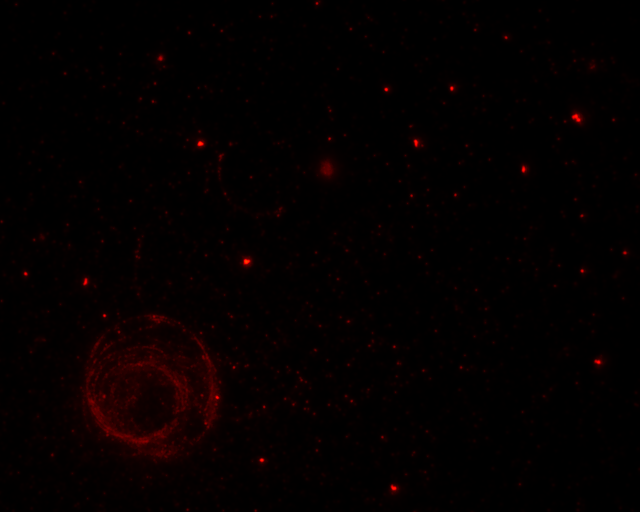

Supplement: Supplementary file 14 — Source data Fig. 7 [file 44318_2025_551_MOESM14_ESM.zip › SD_Figure7_Rev2/Figure7B_ImageData/Figure7B_23-2_Control_Fluorescence.tif]

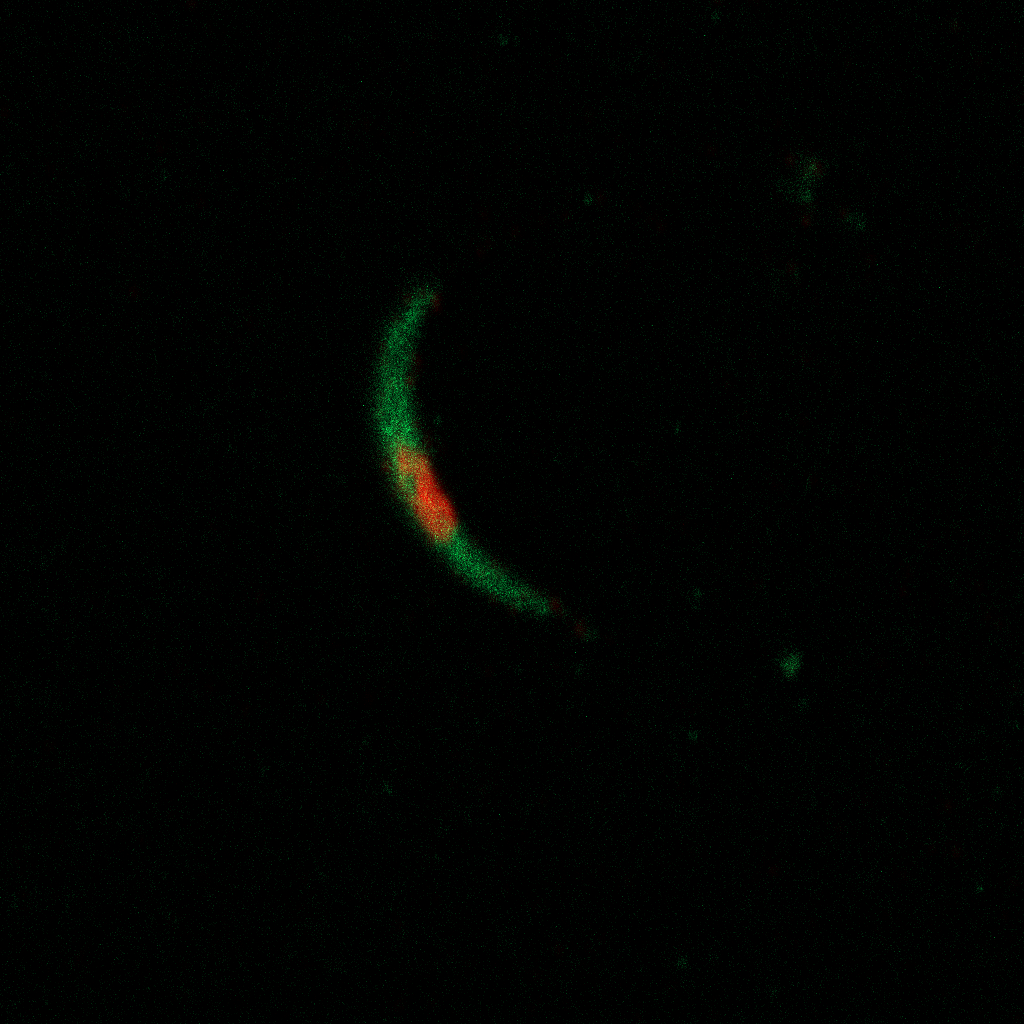

Supplement: Supplementary file 14 — Source data Fig. 7 [file 44318_2025_551_MOESM14_ESM.zip › SD_Figure7_Rev2/Figure7E_ImageData/Figure7E_Rcon_Merge_1.tif]

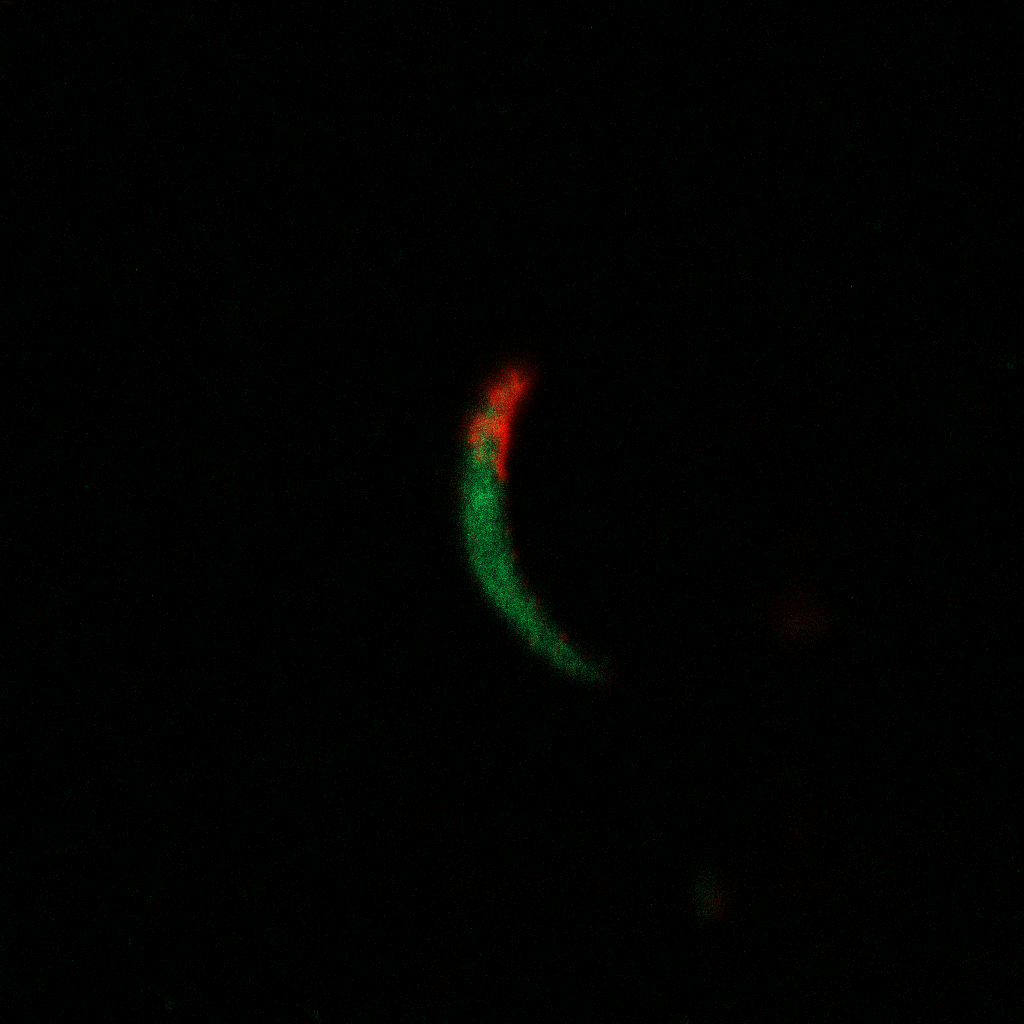

Supplement: Supplementary file 14 — Source data Fig. 7 [file 44318_2025_551_MOESM14_ESM.zip › SD_Figure7_Rev2/Figure7E_ImageData/Figure7E_Rcon_Merge_2.tif]

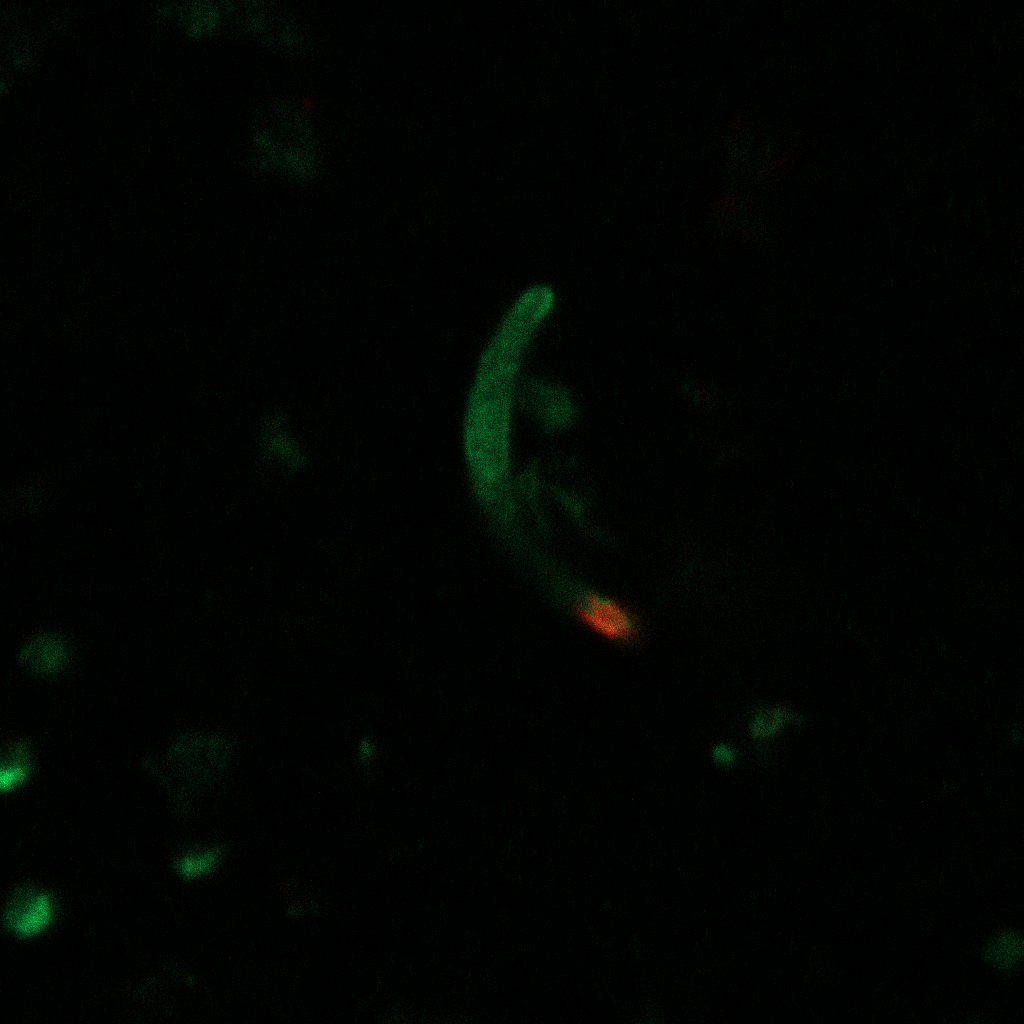

Supplement: Supplementary file 14 — Source data Fig. 7 [file 44318_2025_551_MOESM14_ESM.zip › SD_Figure7_Rev2/Figure7E_ImageData/FIgure7E_Rep2_Merge_1.tif]

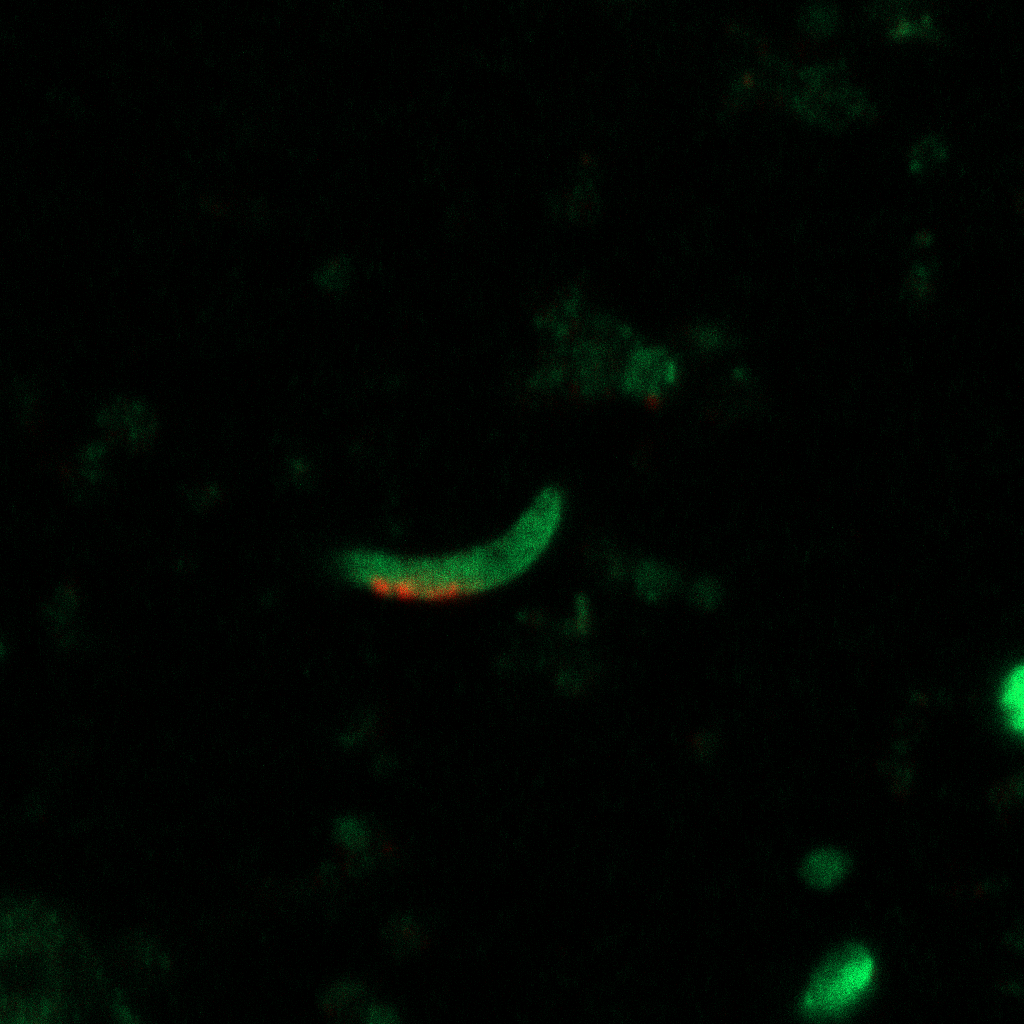

Supplement: Supplementary file 14 — Source data Fig. 7 [file 44318_2025_551_MOESM14_ESM.zip › SD_Figure7_Rev2/Figure7E_ImageData/Figure7E_Rep2_Merge_2.tif]

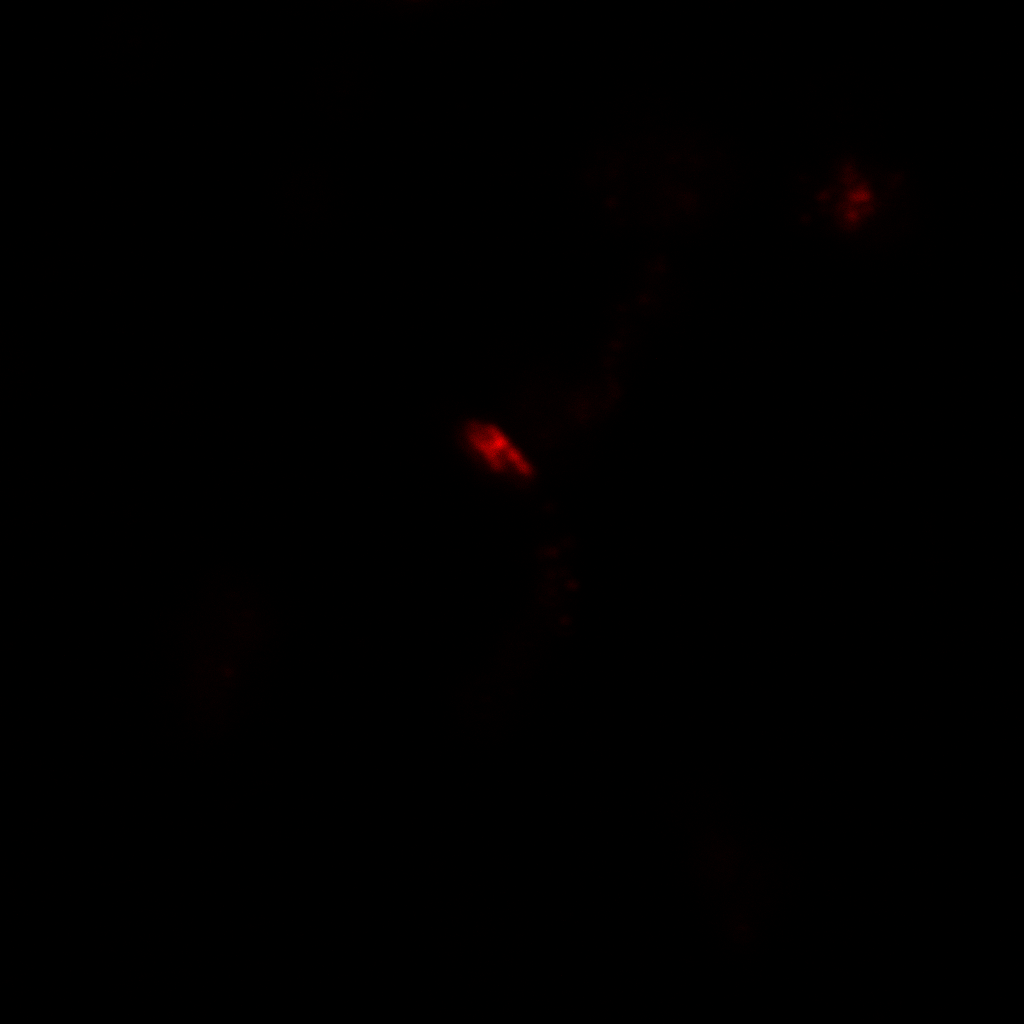

Supplement: Supplementary file 14 — Source data Fig. 7 [file 44318_2025_551_MOESM14_ESM.zip › SD_Figure7_Rev2/Figure7E_ImageData/Figure7E_Scr_MTIP_1.tif]

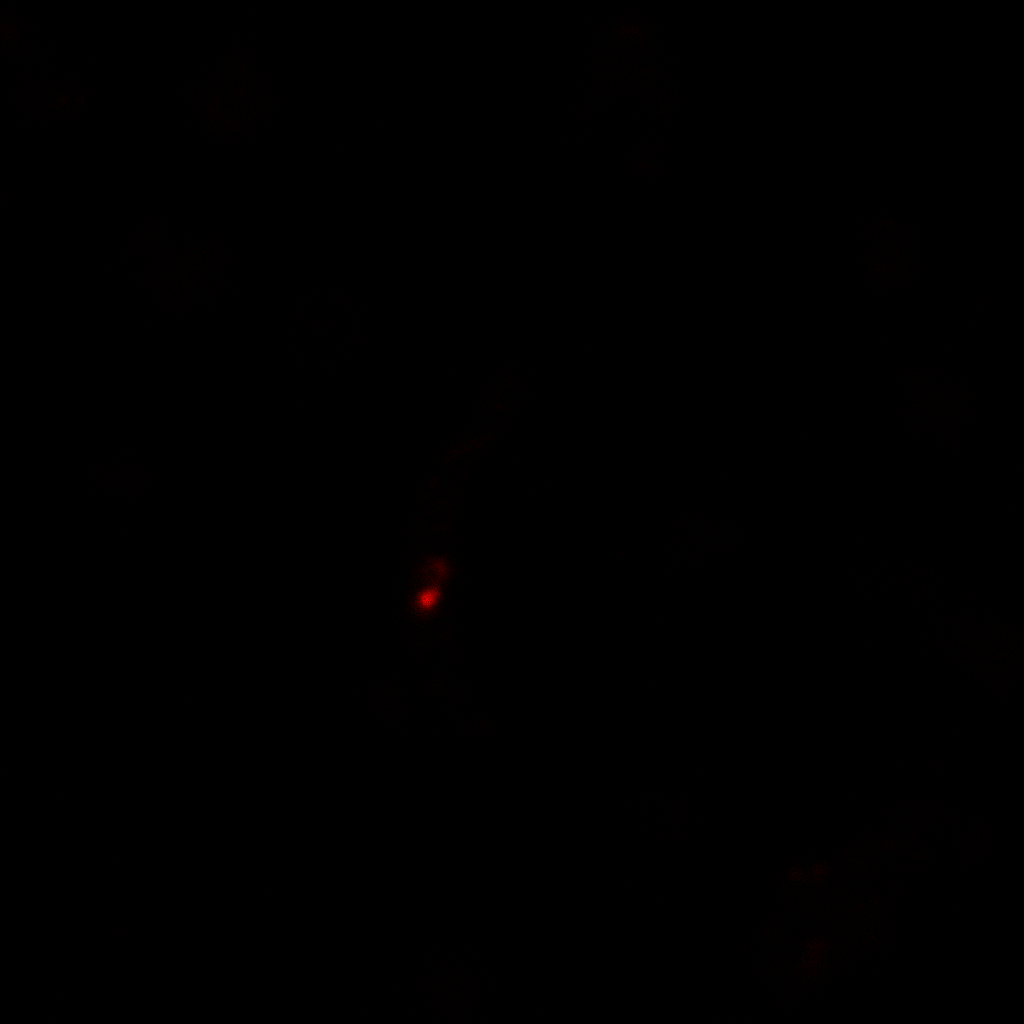

Supplement: Supplementary file 14 — Source data Fig. 7 [file 44318_2025_551_MOESM14_ESM.zip › SD_Figure7_Rev2/Figure7E_ImageData/Figure7E_Scr_MTIP_2.tif]

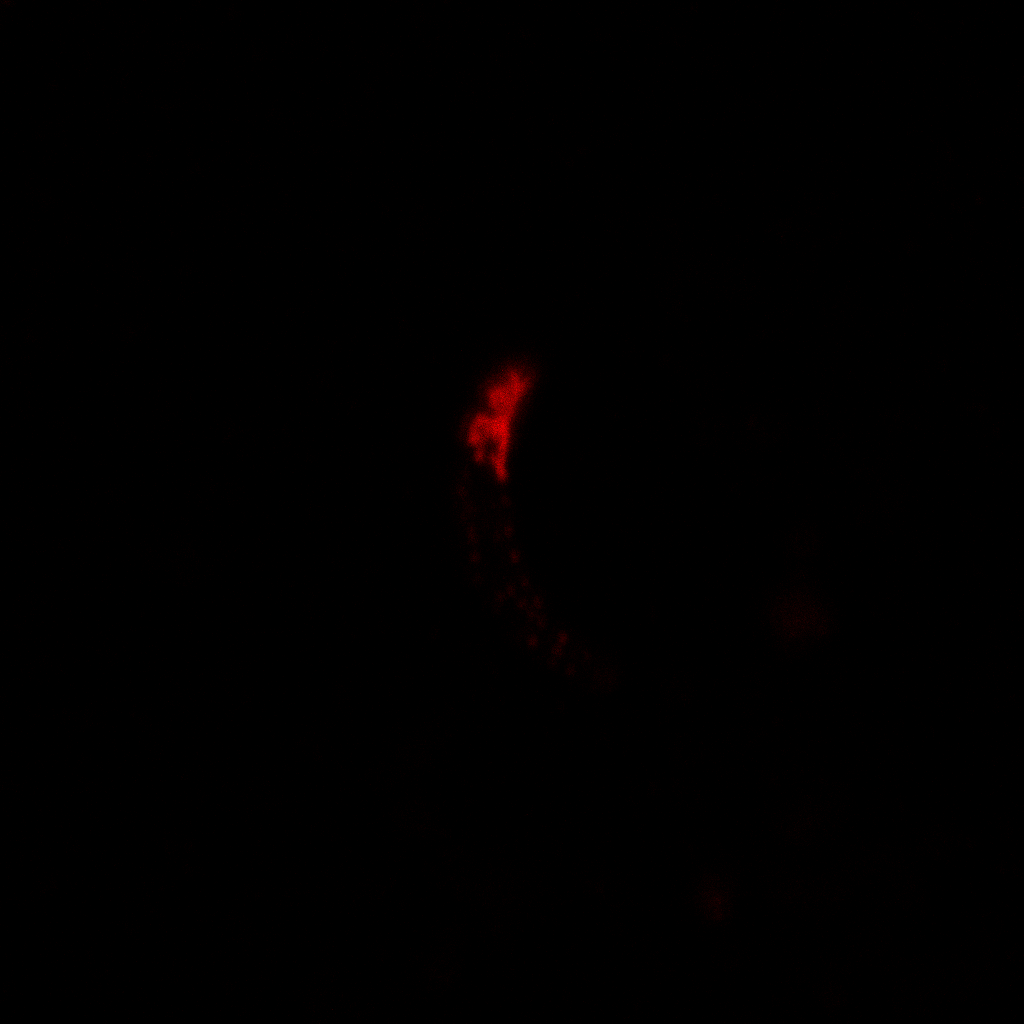

Supplement: Supplementary file 14 — Source data Fig. 7 [file 44318_2025_551_MOESM14_ESM.zip › SD_Figure7_Rev2/Figure7E_ImageData/Figure7E_Rcon_MTIP_2.tif]

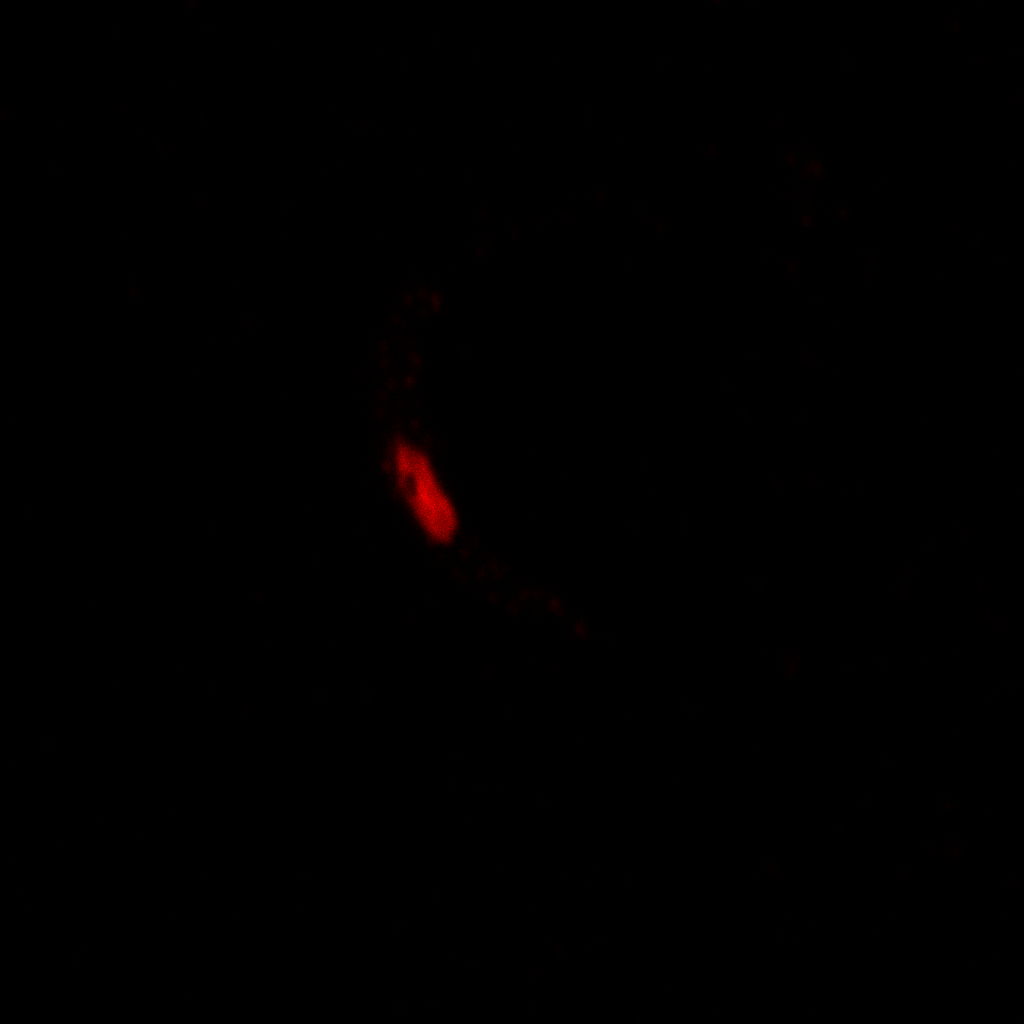

Supplement: Supplementary file 14 — Source data Fig. 7 [file 44318_2025_551_MOESM14_ESM.zip › SD_Figure7_Rev2/Figure7E_ImageData/Figure7E_Rcon_MTIP_1.tif]

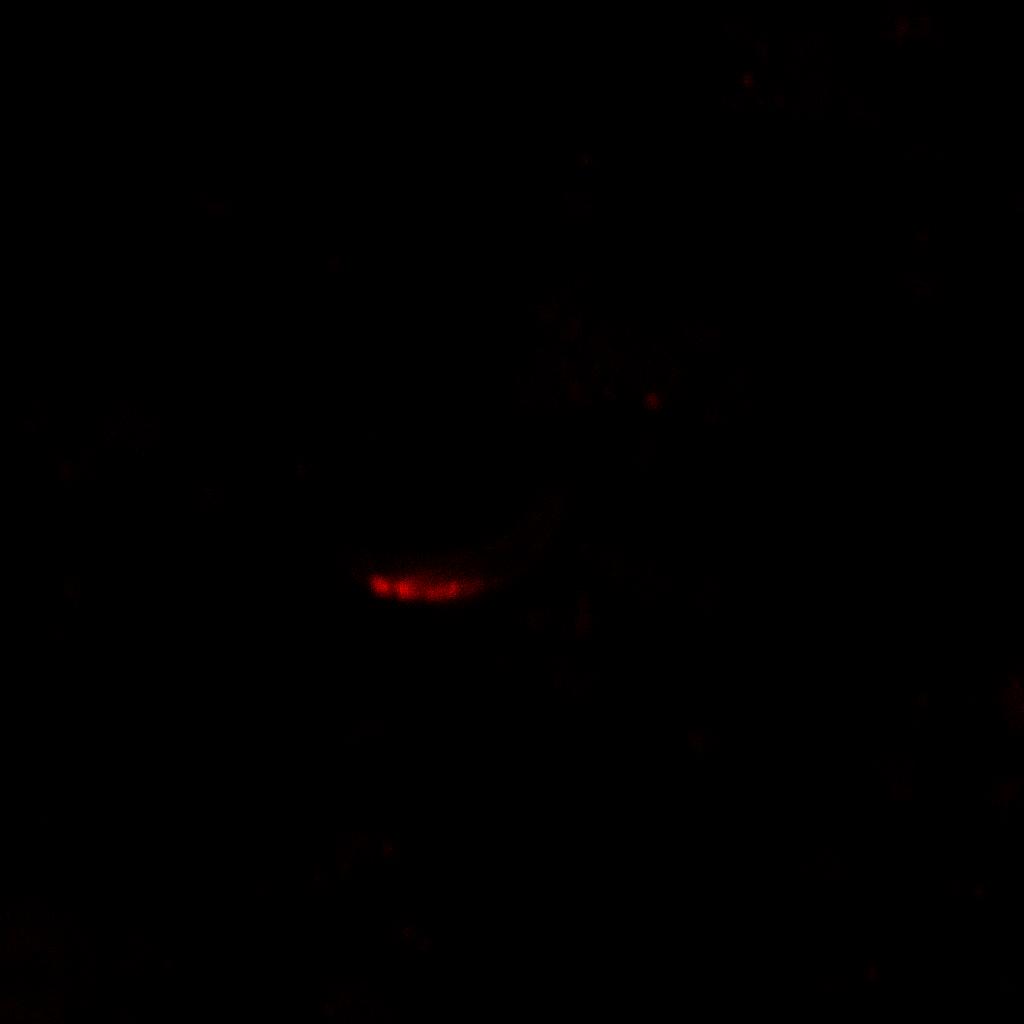

Supplement: Supplementary file 14 — Source data Fig. 7 [file 44318_2025_551_MOESM14_ESM.zip › SD_Figure7_Rev2/Figure7E_ImageData/Figure7E_Rep2_MTIP_2.tif]

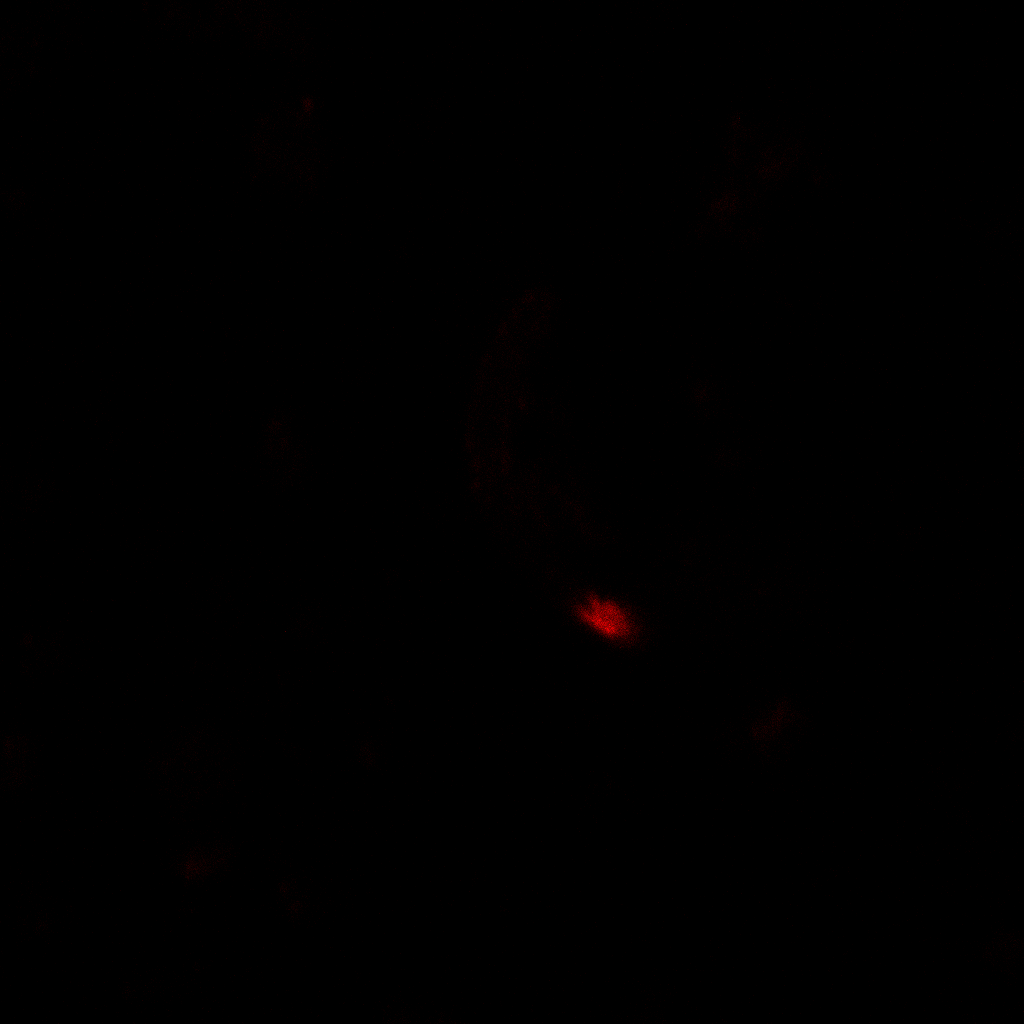

Supplement: Supplementary file 14 — Source data Fig. 7 [file 44318_2025_551_MOESM14_ESM.zip › SD_Figure7_Rev2/Figure7E_ImageData/Figure7E_Rep2_MTIP_1.tif]

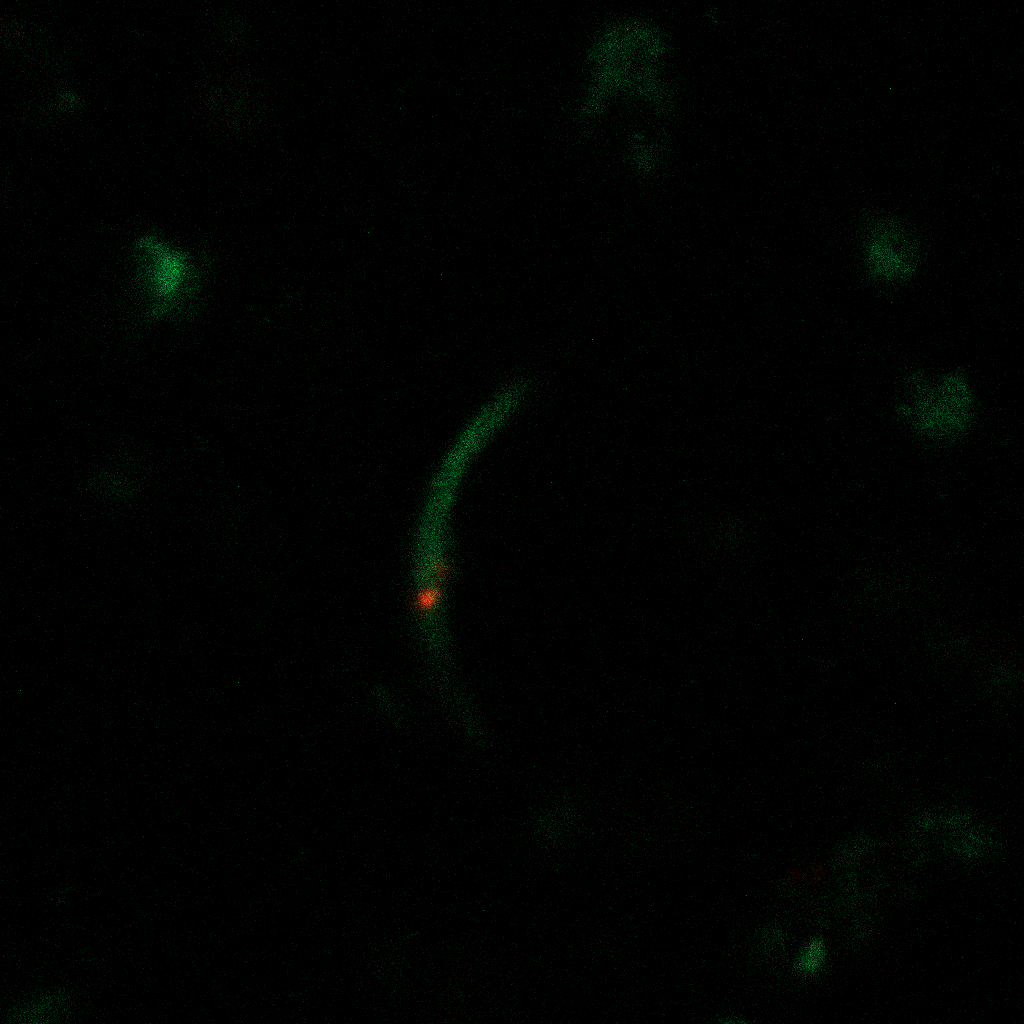

Supplement: Supplementary file 14 — Source data Fig. 7 [file 44318_2025_551_MOESM14_ESM.zip › SD_Figure7_Rev2/Figure7E_ImageData/Figure7E_Scr_Merge_2.tif]

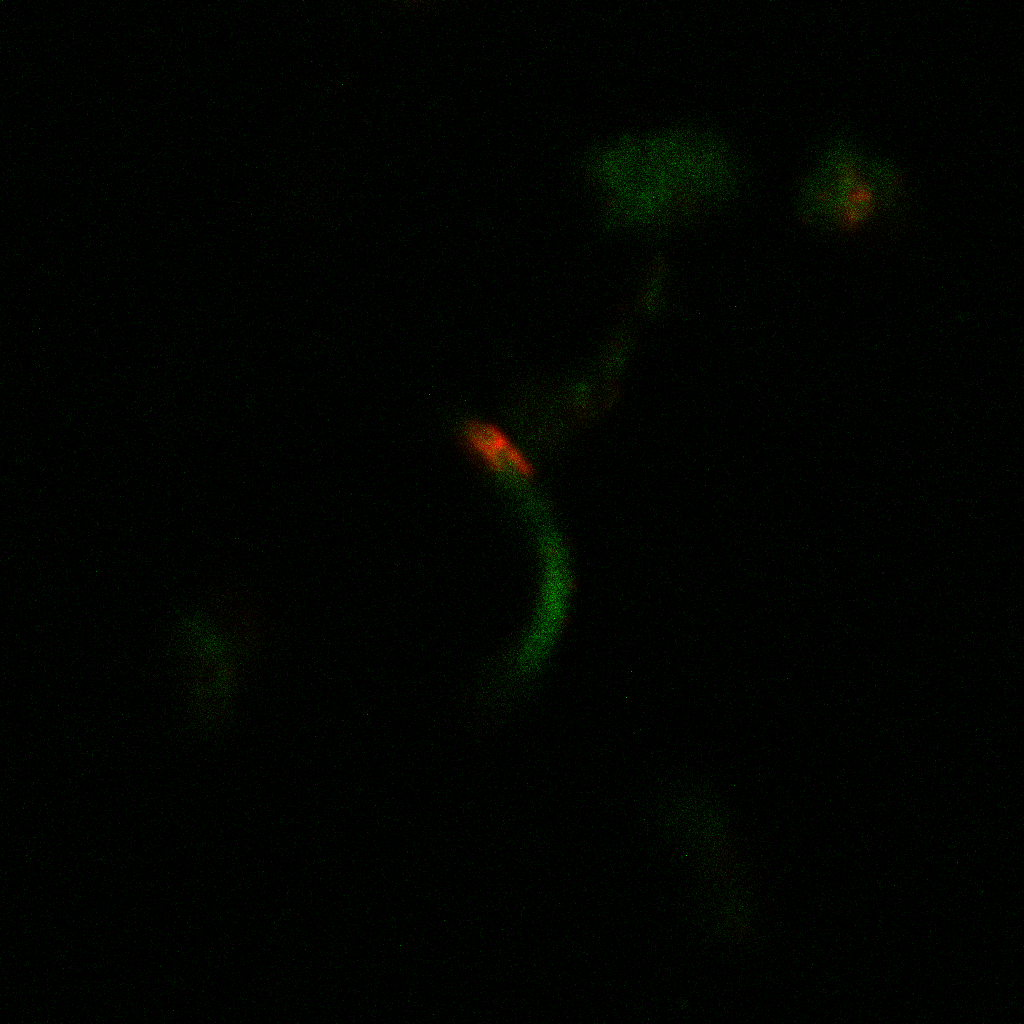

Supplement: Supplementary file 14 — Source data Fig. 7 [file 44318_2025_551_MOESM14_ESM.zip › SD_Figure7_Rev2/Figure7E_ImageData/Figure7E_Scr_Merge_1.tif]

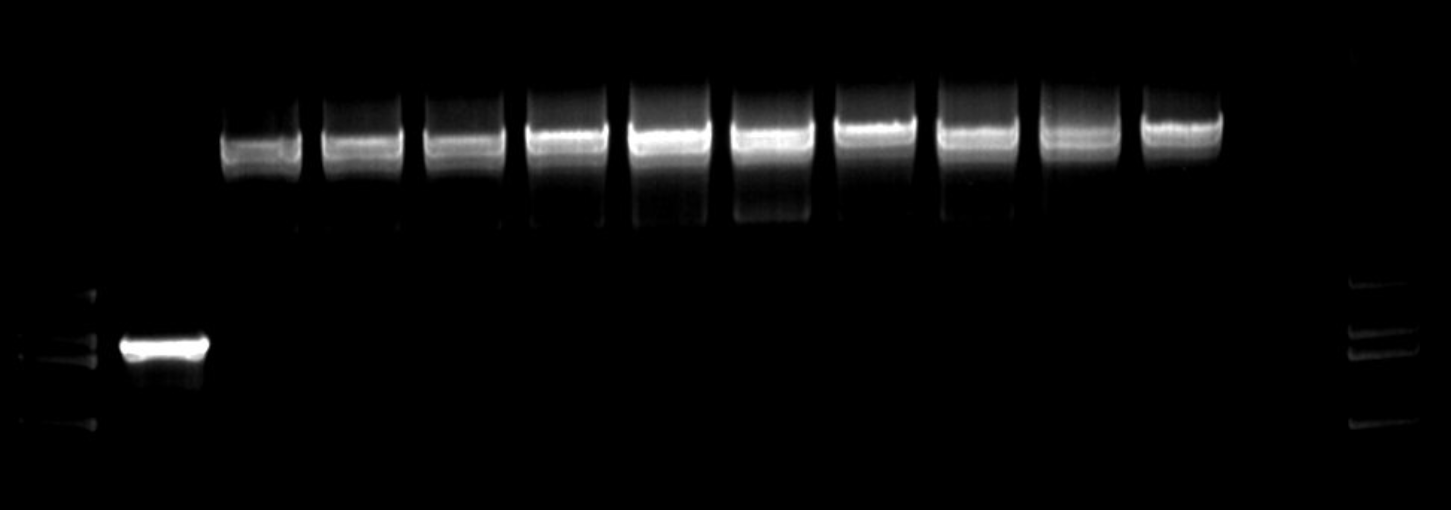

Supplement: Supplementary file 17 — Source data Appendix Figure 1 [file 44318_2025_551_MOESM17_ESM.zip › SuppF1_NewSourceFiles/Size pcr_05-29-18 .tif]

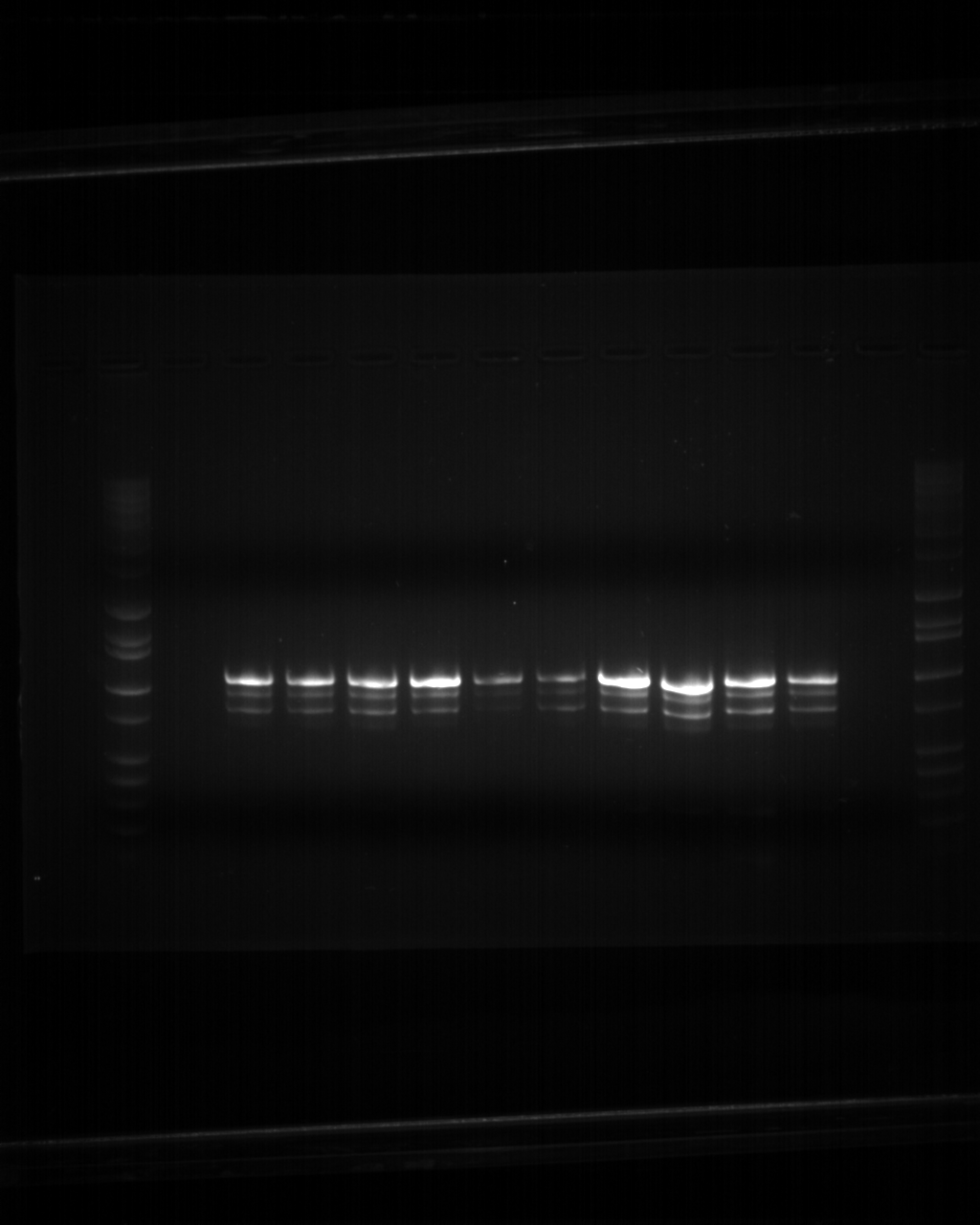

Supplement: Supplementary file 17 — Source data Appendix Figure 1 [file 44318_2025_551_MOESM17_ESM.zip › SuppF1_NewSourceFiles/5 int pcr 2.tif]

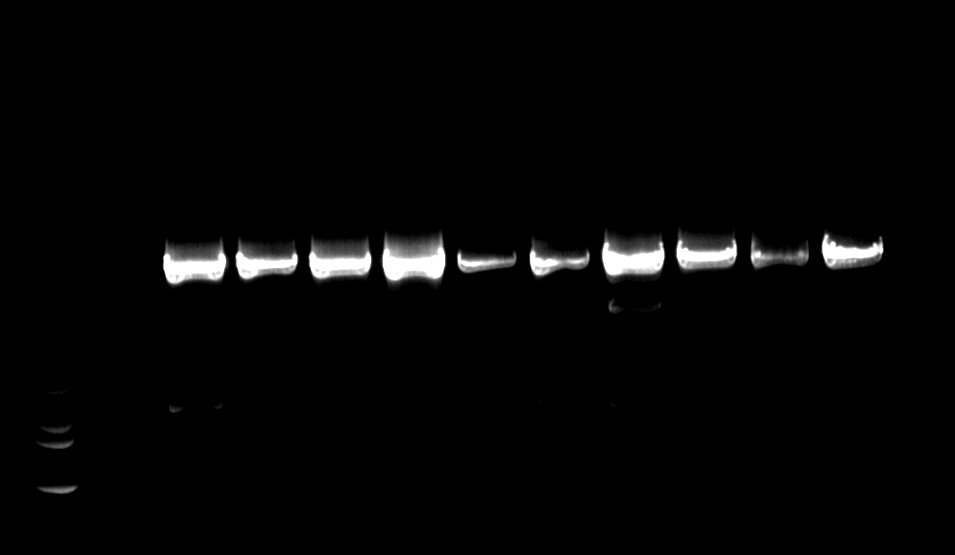

Supplement: Supplementary file 17 — Source data Appendix Figure 1 [file 44318_2025_551_MOESM17_ESM.zip › SuppF1_NewSourceFiles/3'INT_PCR_LowerExp.tif]

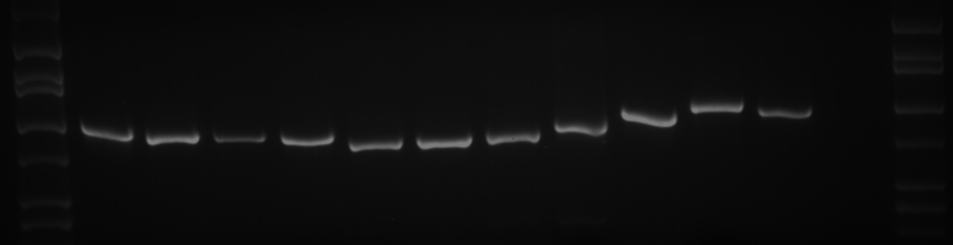

Supplement: Supplementary file 17 — Source data Appendix Figure 1 [file 44318_2025_551_MOESM17_ESM.zip › SuppF1_NewSourceFiles/CSP PCR for paper.tif]
